# Identification of genes expressed in the sex pheromone gland of the black cutworm *Agrotis ipsilon* with putative roles in sex pheromone biosynthesis and transport

**DOI:** 10.1186/1471-2164-14-636

**Published:** 2013-09-22

**Authors:** Shao-Hua Gu, Kong-Ming Wu, Yu-Yuan Guo, John A Pickett, Linda M Field, Jing-Jiang Zhou, Yong-Jun Zhang

**Affiliations:** 1 State Key Laboratory for Biology of Plant Diseases and Insect Pests, Institute of Plant Protection, Chinese Academy of Agricultural Sciences, Beijing 100193, China; 2 Department of Biological Chemistry and Crop Protection, Rothamsted Research, Harpenden AL5 2JQ, UK

## Abstract

**Background:**

One of the challenges in insect chemical ecology is to understand how insect pheromones are synthesised, detected and degraded. Genome wide survey by comparative sequencing and gene specific expression profiling provide rich resources for this challenge. *A. ipsilon* is a destructive pest of many crops and further characterization of the genes involved in pheromone biosynthesis and transport could offer potential targets for disruption of their chemical communication and for crop protection.

**Results:**

Here we report 454 next-generation sequencing of the *A. ipsilon* pheromone gland transcriptome, identification and expression profiling of genes putatively involved in pheromone production, transport and degradation. A total of 23473 unigenes were obtained from the transcriptome analysis, 86% of which were *A. ipsilon* specific. 42 transcripts encoded enzymes putatively involved in pheromone biosynthesis, of which 15 were specifically, or mainly, expressed in the pheromone glands at 5 to 120-fold higher levels than in the body. Two transcripts encoding for a fatty acid synthase and a desaturase were highly abundant in the transcriptome and expressed more than 40-fold higher in the glands than in the body. The transcripts encoding for 2 acetyl-CoA carboxylases, 1 fatty acid synthase, 2 desaturases, 3 acyl-CoA reductases, 2 alcohol oxidases, 2 aldehyde reductases and 3 acetyltransferases were expressed at a significantly higher level in the pheromone glands than in the body. 17 esterase transcripts were not gland-specific and 7 of these were expressed highly in the antennae. Seven transcripts encoding odorant binding proteins (OBPs) and 8 encoding chemosensory proteins (CSPs) were identified. Two CSP transcripts (*AipsCSP2*, *AipsCSP8)* were highly abundant in the pheromone gland transcriptome and this was confirmed by qRT-PCR. One OBP (*AipsOBP6*) were pheromone gland-enriched and three OBPs (*AipsOBP1*, *AipsOBP2* and *AipsOBP4*) were antennal-enriched. Based on these studies we proposed possible *A. ipsilon* biosynthesis pathways for major and minor sex pheromone components.

**Conclusions:**

Our study identified genes potentially involved in sex pheromone biosynthesis and transport in *A. ipsilon*. The identified genes are likely to play essential roles in sex pheromone production, transport and degradation and could serve as targets to interfere with pheromone release. The identification of highly expressed CSPs and OBPs in the pheromone gland suggests that they may play a role in the binding, transport and release of sex pheromones during sex pheromone production in *A. ipsilon* and other Lepidoptera insects.

## Background

Lepidoptera sex pheromones are primarily C10-C18 long straight chain unsaturated alcohols, aldehydes or acetate esters [1], biosynthesised and released mainly from pheromone glands located between the 8^th^ and 9^th^ abdominal segments of the female moths. Usually the females use a mixture of compounds in a unique ratio to attract conspecific males [2]. The extremely high specificity and sensitivity of species-specific pheromones make them potential biological control agents for population monitoring, mass trapping and reducing pesticide use in integrated pest management (IPM) programs [3-5]. Further use of pheromones in such strategies would be aided by an understanding of the pathways involved in pheromone biosynthesis and transport.

Most sex pheromone blends of Lepidoptera insects are synthesised *de novo* via modified fatty acid biosynthesis pathways [2,6,7] and gland-specific enzymes are involved in desaturation, chain shortening, reduction and acetylation [1,2]. Different species use different combinations of these reactions to produce unique species-specific pheromone blends. The first step is the synthesis of saturated fatty acid precursors malonyl-CoA from acetyl-CoA by acetyl-CoA carboxylase (ACC) and fatty acid synthetase (FAS) [8,9]. Labeling studies conducted with acetate indicated that malonyl-CoA and NADPH are used by FAS to produce mainly saturated stearic acid (18:0) and palmitic acid (16:0) with 18 and 16 carbon atoms and no double bonds, respectively, as precursors [10-12]. Modification of the fatty acid chain includes the introduction of a double bond by desaturases specific to pheromone biosynthesis followed by chain shortening using specific β–oxidation enzymes [13,14]. So far, several types of desaturases have been extensively studied through gene characterization and expression analysis, including Δ5 [15], Δ9 [16,17], Δ10 [18], Δ11 [19,20], and Δ14 [21] desaturases. Once unsaturated pheromone precursor with a specific chain-length is produced, the carboxyl carbon is modified to form one of functional groups (aldehyde, alcohol or acetate ester). These modifications require the enzymes fatty acid reductase to produce the alcohols from the fatty acyl precursor [22], which in some species may be oxidized to aldehydes serving as pheromone components [23], and to acetate esters (OAc) by acetyltransferase [24]. Recently, a few members of the reductase gene family have been discovered and functionally characterized in several Lepidoptera species, including *Ostrinia scapulalis*[25], *Heliothis virescens*, *Heliothis subflexa*, *Helicoverpa armigera*, *Helicoverpa assulta*[26], *Ostrinia nubilalis*[27], *Yponomeuta evonymellus* (L.), *Yponomeuta padellus* (L.) and *Yponomeuta rorellus* (Hübner) [28]. A number of pheromone gland-specific enzymes have been identified and their essential functions in pheromone production demonstrated *in vitro* as well as *in vivo*. For example, using RNA interference, Matsumoto and colleagues showed that two pheromone gland-specific enzymes (acyl-CoA desaturase and a fatty-acyl reductase) are responsible for pheromone production in the silk moth *Bombyx mori*[29-31].

After production and release of the sex pheromone components by female moths the males detect the pheromone and respond for mating. It is commonly accepted that pheromone molecules are captured and transported to the pheromone receptors on the dendrites of pheromone-sensitive neurons by olfactory binding proteins, including odorant binding proteins (OBPs) and chemosensory proteins (CSPs) [32-34]. Pheromone binding proteins (PBPs) bind to sex pheromone components and classified into a subclass of OBPs [35]. After activation of the pheromone receptors the olfactory signals must be degraded rapidly to prevent from prolonged neuronal excitation [36]. This may involve pheromone degrading enzymes (PDEs) capable of degrading the pheromone molecules [37].

The black cutworm *Agrotis ipsilon* is a destructive polyphagous insect pest of many crops and for a strain from China the female sex pheromone blend comprises five main acetate components: (*Z*)-11-hexadecenyl acetate (Z11-16:OAc), (*Z*)-9-tetradecenyl acetate (Z9-14:OAc), (*Z*)-7-dodecenyl acetate (Z7-12:OAc), (*Z*)-8-dodecenyl acetate (Z8-12:OAc) and (*Z*)-5-decenyl acetate (Z5-10:OAc) [38]. These components indicate the involvement of different desaturases and ß-oxidases during the sex pheromone biosynthesis. However, the genes/proteins and their specific function in mediating *A. ipsilon* pheromone production, transport and degradation have not been characterized. Over the last few years, the next generation sequencing such as 454 pyrosequencing technique provides an easy and effective method for the discovery of novel genes. In present study, using the Roche GS FLX Titanium sequencing platform, we report a genetic database of the genes expressed in the pheromone glands of *A. ipsilon* and the identification of genes with putative roles in pheromone biosynthesis, degradation and transport as well as their tissue expression profiles.

## Results and discussion

### 454 sequencing and unigene assembly

Sequencing of a cDNA library prepared from mRNAs of the pheromone glands of *A. ipsilon* gave a total of 631,425 raw reads with an average length of 517 base pairs (bp). After trimming adaptor sequences and removing low quality sequences, 629,273 clean reads remained with an average length of 496 bp. The size distribution of the clean reads is shown in Additional file 1. The sequences of all reads have been deposited in the NCBI SRA database with the accession number SRX189143.

The 629,273 clean reads were assembled into 23,473 unigenes, including 20,541 contigs (87.5%) and 2,932 singletons (12.5%), the largest transcriptome dataset so far from moth sex pheromone glands. An overview of the sequencing and assembly results is presented in Table 1. The length of the assembled unigenes ranged from 100 bp to 21842 bp with an average length of 770 bp. Among the unigenes, 22,035 (93.9%) are between 200 bp and 2000 bp long with an average length of 649 bp. These unigenes are in fact transcripts in the *A. ipsilon* pheromone gland cDNA library. Therefore we refer them as transcripts. All sequences of the unigenes used in the current study are provided in Additional file 2.

**Table 1:** **Summary of *A. ipsilon *pheromone gland unigene sequences and assembly**

	**Sequence number**	**Average length (bp)**	**Length distribution(bp)**
Raw reads	631425	517	40-1200 bp
Clean reads	629273	496	40-1200 bp
singletons	2932	421	100-835 bp
contigs	20541	820	100-21842 bp
unigenes	23473	770	100-21842 bp

### Analysis of the transcripts from the *A. ipsilon* pheromone gland

**Figure 1:**
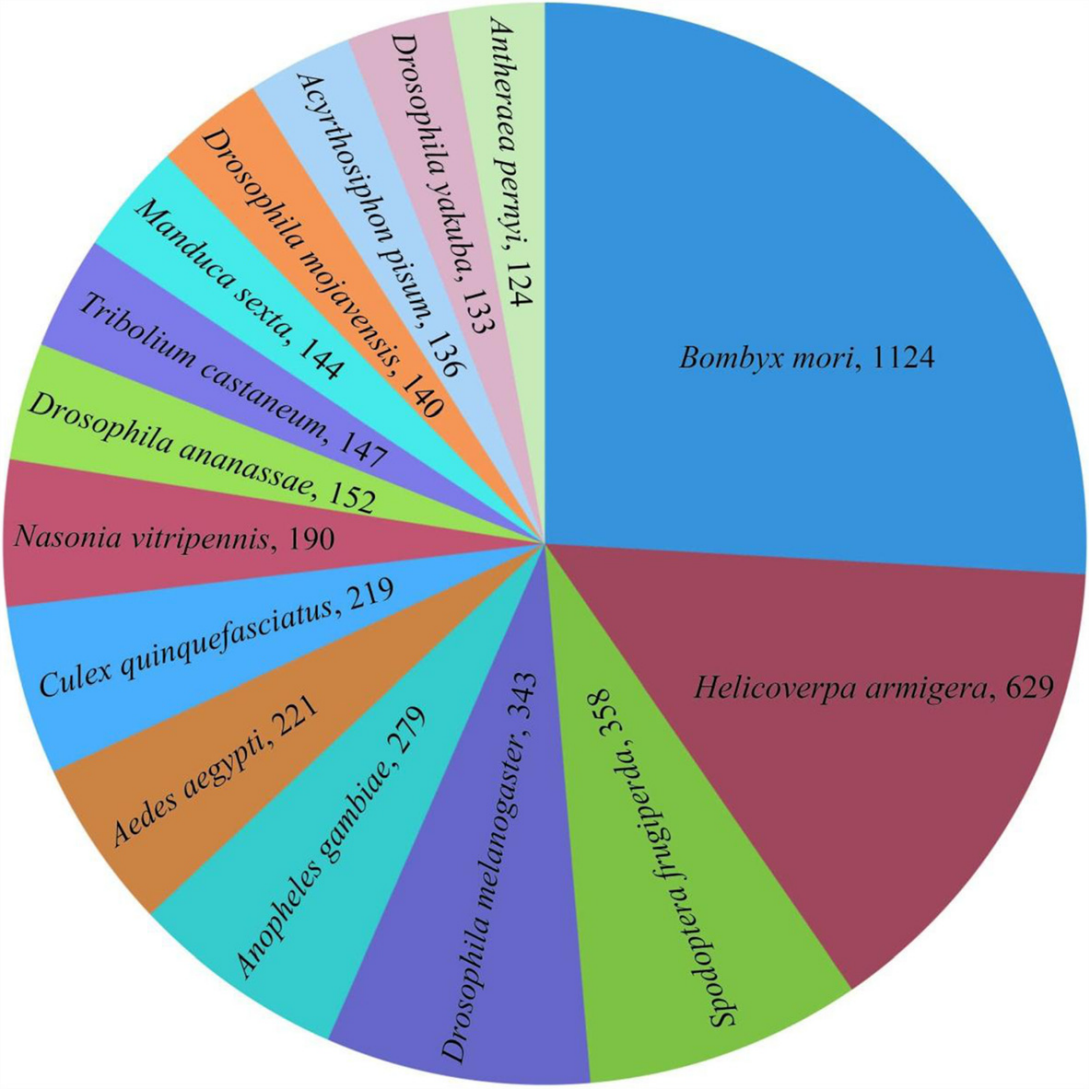
**Top 15 insect species that have significant BLASTn hits.** All *A. ipsilon* pheromone gland unigenes were used in BLASTn searches against the GenBank entries. The significant hits with an E-value >=1.0E-5 for each query were grouped according to species and the number of the unigenes that had significant homology is indicated after the specie name.

BLASTx and BLASTn were used to compare each *A. ipsilon* transcript with a cut-off E-value of 1.0E-5 against GeneBank entries. 12,989 transcripts (55%) had BLASTx hits in the non-redundant protein (nr) databases and 9,392 (40%) had BLASTn hits in the non-redundant nucleotide sequence (nt) databases. This is consistent with a previous report of *H. virescens* pheromone gland ESTs [39]. Some of the *A. ipsilon* transcripts were homologous to those from more than one species but in general most were homologous to other Lepidoptera species taking up 2,379 in the 9,392 BLASTn hits, including 1,124 (12%) to *B. mori* entries. The second highest hits were to Dipteran species with 343 hits to *D. melanogaster* and 279 and 221 hits to the mosquitoes *Anopheles gambiae* and *Aedes aegypti*, respectively. The lowest hits were to the wasp *Nasonia vitripennis* (190 hits), the beetle *Tribolium castaneum* (147 hits) and the pea aphid *Acyrthosiphon pisum* (136 hits). The top 15 insect species that have significant BLASTn hits are shown in Figure 1.

### Gene Ontology of the genes expressed in the *A. ipsilon* pheromone gland

The 23,473 assembled transcripts were annotated into different functional groups according to Gene Ontology (GO) analysis. Some transcripts were annotated into more than one GO category. Of the 22,473 transcripts, 7,546 (32%) could be assigned to a GO category (Additional file 3). The “cellular process” and “metabolic process” GO categories were most abundantly represented with 4,056 (17.3%) and 3,361 (14.3%) transcripts, respectively, within the biological process GO ontology. In the “cellular components” GO ontology the transcripts were mainly distributed in cell (18.8%) (4,415 transcripts) and cell part (17.6%) (4,133 transcripts). The GO analysis also showed that in the molecular function ontology 3,271 transcripts (13.9%) were annotated as having binding functions and 3,484 (14.8%) to have catalytic activity.

### Comparative analysis of transcripts in Lepidoptera pheromone glands

In order to compare the *A. ipsilon* pheromone gland transcriptome with those from other Lepidoptera and to identify *A. ipsilon* transcripts with potential involvement in sex pheromone production and transport we downloaded the pheromone gland ESTs of three other Lepidoptera *A. segetum*, *B. mori* and *H. virescens* from the dbEST database of NCBI and previously published pheromone gland transcriptome of *H. virescens*[39]. After assembling these ESTs we obtained 925 unigenes from *A. segetum*, 3943 from *B. mori* and 8202 from *H. virescens* with an average length of 384 bp, 692 bp and 474 bp, respectively. These are much lower numbers than that obtained by the current study through the 454 sequencing of the *A. ipisilon* pheromone gland, demonstrating that our pheromone gland transcriptome is currently the largest transcriptome resource for an insect pheromone gland.

**Figure 2:**
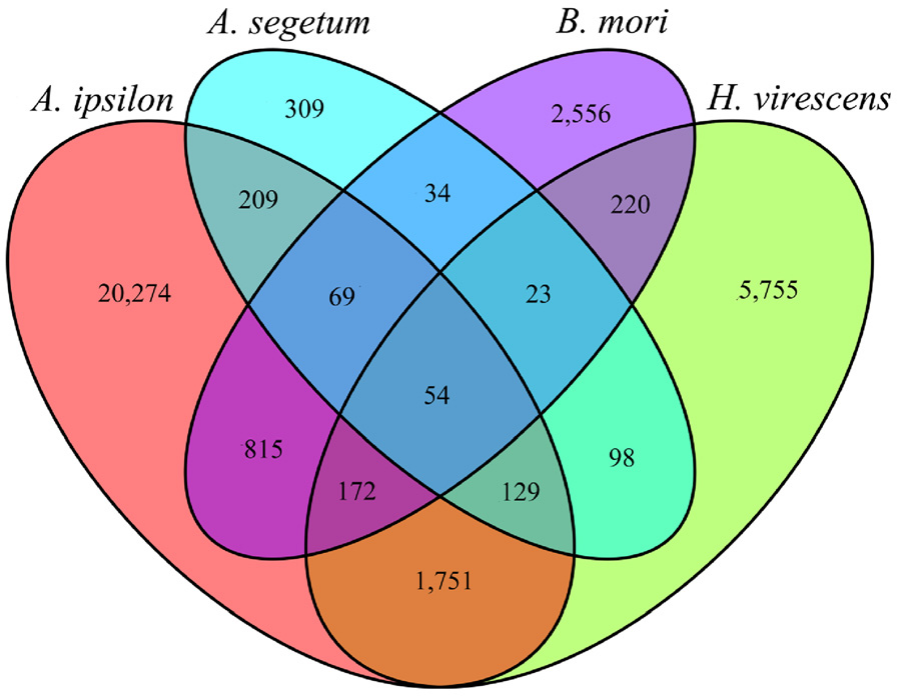
**Comparative analysis of *A. ipsilon *pheromone gland unigenes with other insects. This shows the overlap of blast homology in genes expressed in pheremone glands in four species of Lepidoptera. **The comparative analyses of *A. ipsilon*, *H. virescens*, *B. mori* and *A. segetum* pheromone gland unigenes were performed based on the Best Bidirectional Hits results (reciprocal BLASTn, E-value less than 1.0E-6).

When comparing the pheromone gland transcripts pairwise using best bidirectional hits, we found that there were 461 homologous transcripts between *A*. *ipsilon* and *A. segetum*, 1110 homologous transcripts between *A. ipsilon* and *B. mori*, and 2106 homologous transcripts between *A. ipsilon* and *H. virescens* (Figure 2). A large portion of *A. ipsilon* transcripts (86.4%) (20,274 out of 23,473) had no homologous ESTs in the available pheromone gland EST libraries of the other 3 species. This may be due to the larger dataset (23,473 unigenes) for *A. ipsilon* and lower coverage in the other studies. Nevertheless, it was shown that 309 transcripts, 5,755 transcripts and 2,556 transcripts are only found in *A. segetum, H. virescen* and *B. mori*, respectively, in our comparison (Figure 2).

### Transcript abundance in the *A. ipsilon* pheromone gland

The pheromone gland mRNA samples used for constructing the cDNA library were non-normalized and non-amplified by PCR, so the reads in the sequencing dataset most likely represent the relative abundance of each assembled transcript in the pheromone gland as summarized in Table 2. The most abundant transcripts include vitellogenin, a major reproductive protein in insects (2,925 reads per kilobase per million mapped reads (RPKM); 2.2% reads), the precursor of egg yolk proteins for insect egg production [40] and genes involved in PBAN stimulated pheromone production such as lipase 3 [41] (4,731 RPKM; 0.8% reads) and in sex pheromone biosynthesis such as acyl-CoA desaturase (1,206 RPKM; 0.3% reads) and in lipid transport such as apolipophorin III (2894 RPKM 0.4% reads). Another highly abundant transcript (*Unigene_721*) with 1,365 RPKM encodes a CSP with a 76% protein identity to the *H. virescens* CSP (Protein ID: ACX53806) and 41% to the ejaculatory bulb-specific protein 3 of *D. melanogaster* (Protein ID: Q9W1C9).

**Table 2:** **The most prevalent mRNAs in *A. ipsilon *sex pheromone gland**

**Unigene ID**	**GenBank annotation**	**Species**	**Accession number**	**Score**	***E*-value**	**% Identity**	**No. of Reads**
Unigene_694	vitellogenin	*Spodoptera litura*	ABU68426	2195	0.0	71%	10106
Unigene_3480	lipase 3	*Danaus plexippus*	EHJ71865	344	5E-108	47%	5201
Unigene_17140	C-type lectin 5	*Helicoverpa armigera*	AFI47450	411	4E-141	62%	4131
Unigene_2237	vitellogenin	*Spodoptera litura*	ABU68426	673	0.0	66%	3500
Unigene_688	translation elongation factor 2	*Spodoptera exigua*	AAL83698	1565	0.0	96%	3122
Unigene_18106	apolipophorin precursor	*Bombyx mori*	BAK82317	4076	0.0	61%	2457
Unigene_692	apolipophorin precursor	*Manduca sexta*	AAB53254	2536	0.0	63%	2274
Unigene_17571	calcium ATPase	*Heliothis virescens*	AAD09820	1868	0.0	97%	2048
Unigene_2742	ADP/ATP translocase	*Helicoverpaarmigera*	AAP20934	397	2E-136	92%	2039
Unigene_17691	heat shock e protein 70	*Agrotis ipsilon*	AEG78288	1131	0.0	100%	1832
Unigene_728	cathepsin	*Helicoverpa armigera*	NP_075125	263	9E-82	43%	1783
Unigene_7978	elongation factor 1 alpha	*Papili opolytes*	BAM18878	895	0.0	99%	1769
Unigene_178	apolipophorin III	*Trichoplusia ni*	ABV68867	288	2E-96	88%	1709
Unigene_780	acyl-CoA desaturase	*Helicoverpa assulta*	AF482909	692	0.0	94%	1691
Unigene_3446	myosin regulatory light chain 2	*Bombyx mori*	NP_001091813	298	1E-99	98%	1627
Unigene_18120	fatty acid synthase	*Tribolium castaneum*	XP_970417	2436	0.0	57%	1486
Unigene_721	Chemosensory protein	*Heliothis virescens*	ACX53806	206	3E-66	76%	390

### Candidate genes in the *A. ipsilon* pheromone gland with putative functions in pheromone production, transport and degradation

**Figure 3:**
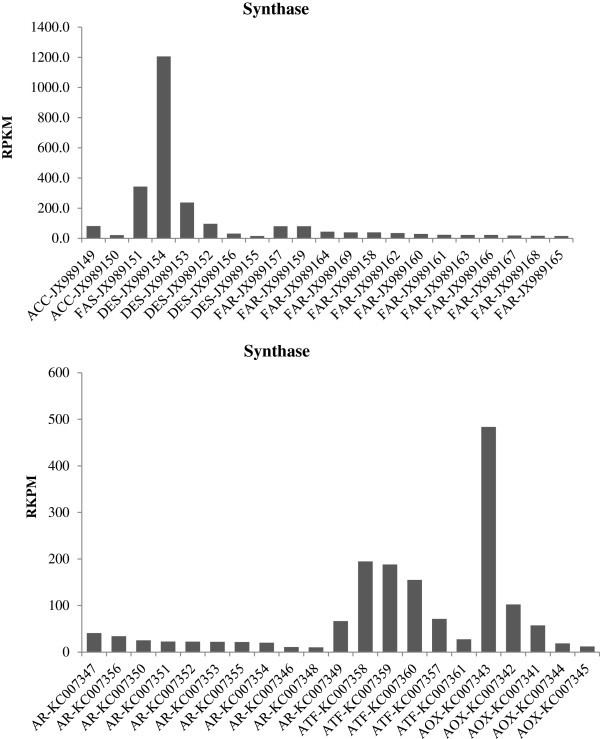
**The abundance of the unigenes encoding the sex pheromone synthase in the *A. ipsilon *transcriptome dataset presented as normalized read count in reads per kilobase per million mapped reads (RPKM).** The putative enzyme names are indicated as gene abbreviations followed by Genbank accession numbers. *ACC* Acetyl-CoA carboxylase, *AOX* Alcohol oxidase, *AR* Aldehyde reductase, *ATF* Acetyltransferase, *DES* Desaturase, *FAR* Fatty acyl reductase, *FAS* Fatty acid synthase.

**Figure 4:**
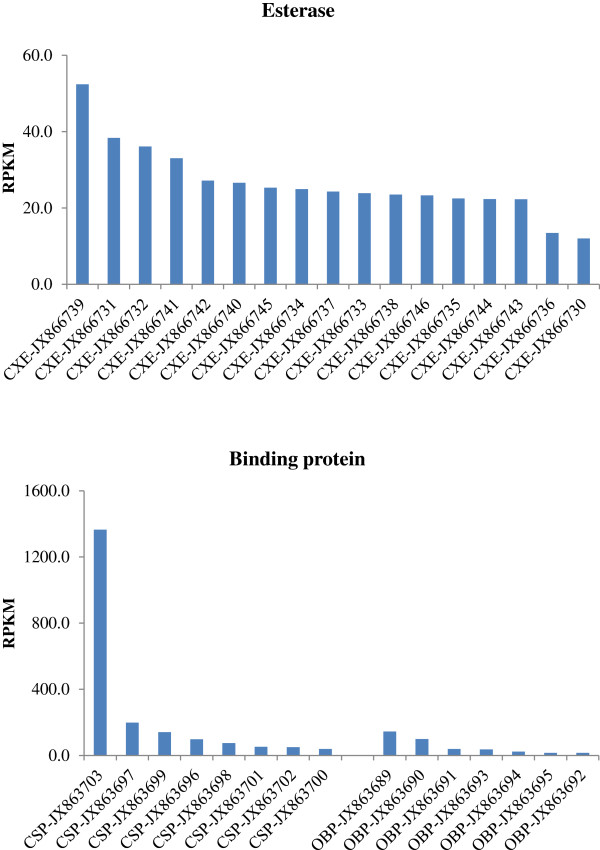
**The abundance of unigenes encoding chemosensory proteins (CSPs), odorant-binding proteins (OBPs) and esterase (EST) in the *A. ipsilon *transcriptome dataset presented as normalized reads in reads per kilobase per million mapped reads (RPKM).**

The overall enzymatic steps during pheromone biosynthesis in *A. ipsilon* are likely to be similar to those in other moth species, which include fatty acid synthesis, desaturation, chain shortening, reduction and acetylation [1,2,6]. By homologous searches we identified members of gene subfamilies in the *A. ipsilon* pheromone gland transcriptome putatively involved in these biosynthetic processes and pheromone production, including transcripts putatively encoding 3 synthases (2 actyl-CoA carboxylase and 1 fatty acid synthase), 5 desaturases, 13 acyl-CoA reductases, 5 alcohol oxidases and 5 acetyltransferases as well as 11 aldehyde reductases (Table 3); 17 transcripts encoding putative pheromone degradation enzymes (Table 4); 8 transcripts encoding putative CSPs and 7 transcripts encoding putative OBPs (Table 5). Their abundances in the pheromone gland transcriptome are shown in Figures 3 and 4. We further validated and characterized the expression level and the tissue distribution of these genes by RT-PCR and qRT-PCR and summarised below. There is a clear agreement between the transcript abundance estimated by the transcriptome sequencing and transcript expression level in the pheromone gland as measured by RT-PCR and qRT-PCR.

**Table 3:** **Putative pheromone biosynthesis related genes in the *A. ipsilon *pheromone gland**

Unigene*	**Accession Num.**	**Length (bp)**	**No. of Reads**	**Putative identification**	**Species**	**Accession Num.**^**§**^	**Score (bits)**	***E*-value**	**% Identity**
**Acetyl CoA Carboxylase**									
Unigene_2338-ACC	JX989149	7534	305	acetyl-CoA carboxylase	*Tribolium castaneum*	XP_969851	2644	0.0	67%
Unigene_6244-ACC	JX989150	621	17	acetyl-CoA carboxylase	*Heliothis virescens*	ACX53705	169	2E-47	56%
**Fatty acid synthase**									
Unigene_18120-FAS	JX989151	8703	2341	fatty acid synthase	*Tribolium castaneum*	XP_970417	2436	0.0	57%
**Desaturases**									
Unigene_65-DES	JX989152	1077	154	acyl-CoA delta 9 desaturase	*Mamestra brassicae*	ABX90048	681	0.0	96%
Unigene_741-DES	JX989153	1047	630	acyl-CoA delta 11 desaturase	*Mamestra brassicae*	ABX90049	610	0.0	87%
Unigene_780-DES	JX989154	1077	1710	acyl-CoA desaturase HassNPVE	*Helicoverpa assulta*	AF482909	692	0.0	94%
Unigene_10494-DES	JX989155	254	3	desaturase	*Spodoptera littoralis*	AAQ74260	98.2	3E-22	64%
Unigene_15401-DES	JX989156	489	9	acyl-CoA desaturaseSexiGATD	*Spodoptera exigua*	AAM28510	323	1E-110	93%
**Fatty acyl reductase**									
Unigene_163-FAR	JX989157	1593	82	fatty-acyl CoA reductase 6	*Ostrinia nubilalis*	ADI82779	536	0.0	59%
Unigene_1098-FAR	JX989158	948	43	putative fatty acyl-CoA reductase	*Nasonia vitripennis*	XP_001600309	261	2E-119	51%
Unigene_2537-FAR	JX989159	1560	105	putative fatty acyl-CoA reductase	*Bombus terrestris*	XP_003399879	585	0.0	57%
Unigene_3905-FAR	JX989160	1593	37	putative fatty acyl-CoA reductase	*Apis mellifera*	ADI87410	541	0.0	59%
Unigene_4078-FAR	JX989161	1017	14	fatty-acyl CoA reductase 3	*Ostrinia nubilalis*	ADI82776	519	2E-178	80%
Unigene_4302-FAR	JX989162	861	56	fatty-acyl CoA reductase 6	*Danaus plexippus*	EHJ76493	372	8E-174	61%
Unigene_6708-FAR	JX989163	615	7	fatty-acyl CoA reductase 4	*Ostrinia nubilalis*	ADI82777	342	7E-113	77%
Unigene_7344-FAR	JX989164	1380	43	fatty-acyl reductase	*Heliothis virescens*	ACX53790	671	0.0	70%
Unigene_8541-FAR	JX989165	375	4	fatty-acyl CoA reductase 5	*Ostrinia nubilalis*	ADI82778	193	4E-63	69%
Unigene_11561-FAR	JX989166	182	2	fatty-acyl-CoA reductase	*Heliothis virescens*	ACX53773	104	2E-26	82%
Unigene_12329-FAR	JX989167	855		fatty-acyl CoA reductase 6	*Ostrinia nubilalis*	ADI82779	315	3E-101	66%
Unigene_12652-FAR	JX989168	249	2	fatty-acyl-CoA reductase	*Heliothis virescens*	ACX53773	117	5E-31	76%
Unigene_15351-FAR	JX989169	714	14	putative fatty acyl-CoA reductase	*Apis mellifera*	ADI87410	283	6E-90	56%
Unigene	Accession Num.	Length (bp)	No. of Reads	Putative identification	Species	Accession Num.^§^	Score (bits)	*E*-value	% Identity
**Alcohol oxidase**									
Unigene_195-AOX	KC007341	834	25	Putative alcohol dehydrogenase	*Danaus plexippus*	EHJ70611	221	2E-67	43%
Unigene_307-AOX	KC007342	969	56	Putative alcohol dehydrogenase	*Danaus plexippus*	EHJ70611	327	3E-106	55%
Unigene_397-AOX	KC007343	756	253	alcohol dehydrogenase	*Heliothis virescens*	ACX53694	405	6E-139	78%
Unigene_7733-AOX	KC007344	576	6	Putative alcohol dehydrogenase	*Danaus plexippus*	EHJ70611	219	7E-68	64%
Unigene_10714-AOX	KC007345	501	3	Putative alcohol dehydrogenase	*Danaus plexippus*	EHJ73729	230	8E-72	68%
**Aldehyde reductase**									
Unigene_12563-AR	KC007346	849	45	putative aldo-ketosereductase 1	*Papilio dardanus*	CAW30924	394	2E-134	70%
Unigene_1274-AR	KC007347	945	34	aldo-ketoreductase	*Bombyx mori*	ADQ89807	491	2E-168	78%
Unigene_17351-AR	KC007348	930	43	aldo-ketoreductase 2E	*Bombyx mori*	BAL70378	342	1E-113	58%
Unigene_1774-AR	KC007349	954	37	aldo-ketoreductase	*Helicoverpa armigera*	AEB26313	577	0.0	88%
Unigene_3134-AR	KC007350	975	35	putative aldo-ketoreductase	*Danaus plexippus*	EHJ71186	410	9E-140	66%
Unigene_4806-AR	KC007351	726	9	aldehyde reductase 1	*Culex quinquefasciatus*	XP_001844836	302	1E-98	64%
Unigene_5103-AR	KC007352	598	8	aldo-ketoreductase	*Heliothis virescens*	ACX53715	296	2E-96	72%
Unigene_7337-AR	KC007353	456	5	aldo-ketoreductase	*Heliothis virescens*	ACX53798	236	2E-74	80%
Unigene_7554-AR	KC007354	552	92	aldo-ketoreductase, partial	*Papilio xuthus*	BAM20078	183	4E-54	57%
Unigene_9245-AR	KC007355	645	8	aldo-ketoreductase	*Papilio xuthus*	BAM19656	233	8E-73	53%
Unigene_9786-AR	KC007356	963	28	aldo-ketoreductase	*Danaus plexippus*	EHJ68075	407	3E-135	63%
**Acetyltransferase**									
Unigene_173-ATF	KC007357	795	43	acetyltransferase 1	*Danaus plexippus*	EHJ65205	317	1E-145	71%
Unigene_407-ATF	KC007358	855	196	acyltransferase	*Heliothis virescens*	ACX53812	494	5E-172	90%
Unigene_553-ATF	KC007359	1038	141	putative acetyl transferase	*Bombyx mori*	NP_001182381	528	0.0	86%
Unigene_2015-ATF	KC007360	552	67	acetyltransferase	*Danaus plexippus*	EHJ65977	327	6E-110	90%
Unigene_15362-ATF	KC007361	1010	69	putative acetyl-CoA acetyltransferase	*Danaus plexippus*	EHJ68573	444	3E-150	74%

*Gene names as deposited in GenBank.^§^Genbank accession number of the homologous gene. *ACC* Acetyl-CoA carboxylase, *FAS* Fatty acid synthase, *DES* Desaturase, *FAR* Fatty acyl reductase, *AOX* alcohol oxidase, *AR* Aldehyde reductase, *ATF* Acetyltransferase.

**Table 4:** **Candidate esterase genes likely involved in *A. ipsilon *pheromone degradation**

Gene name*	**Unigene**	**Accession num.**	**Length (bp)**	**No. of reads**	**Putative identification**	**Species**	**Accession num.**^**§**^	**Score (bits)**	***E*-value**	**% Identity**
AipsCXE1	Unigene_8856	JX866730	459	4	esterase	*Spodoptera littoralis*	ABH01081	219	9E-83	75%
AipsCXE2	Unigene_1631	JX866731	1148	22	antennal esterase CXE2	*Spodoptera littoralis*	ACV60229	475	5E-161	67%
AipsCXE3	Unigene_1377	JX866732	969	20	antennal esterase CXE3	*Spodoptera littoralis*	ACV60230	518	2E-178	82%
AipsCXE4	Unigene_7378	JX866733	504	6	antennal esterase CXE4	*Spodoptera littoralis*	ACV60231	252	6E-78	77%
AipsCXE5	Unigene_4213	JX866734	1047	13	antennal esterase CXE5	*Spodoptera littoralis*	ACV60232	647	0.0	87%
AipsCXE6	Unigene_4686	JX866735	554	7	antennal esterase CXE6	*Spodoptera littoralis*	ACV60233	229	2E-69	75%
AipsCXE7	Unigene_9837	JX866736	746	5	antennal esterase CXE7	*Spodoptera littoralis*	ACV60234	338	7E-110	70%
AipsCXE8	Unigene_4231	JX866737	907	11	antennal esterase CXE8	*Spodoptera littoralis*	ACV60235	381	5E-126	69%
AipsCXE9	Unigene_7661	JX866738	512	6	antennal esterase CXE9	*Spodoptera littoralis*	ACV60236	226	5E-68	64%
AipsCXE10	Unigene_68	JX866739	1674	54	antennal esterase CXE10	*Spodoptera littoralis*	ACV60237	387	6E-123	41%
AipsCXE11	Unigene_1628	JX866740	336	9	antennal esterase CXE11	*Spodoptera littoralis*	ACV60238	160	1E-74	83%
AipsCXE12	Unigene_4126	JX866741	444	16	antennal esterase CXE12	*Spodoptera littoralis*	ACV60239	192	3E-53	50%
AipsCXE13	Unigene_15183	JX866742	1843	25	antennal esterase CXE13	*Spodoptera littoralis*	ACV60240	974	0.0	83%
AipsCXE14	Unigene_7537	JX866743	451	5	antennal esterase CXE14	*Spodoptera littoralis*	ACV60241	256	5E-80	85%
AipsCXE15	Unigene_5546	JX866744	1380	19	antennal esterase CXE15	*Spodoptera littoralis*	ACV60242	498	5E-167	52%
AipsCXE16	Unigene_7483	JX866745	474	6	antennal esterase CXE16	*Spodoptera littoralis*	ACV60243	268	3E-84	77%
AipsCXE20	Unigene_5325	JX866746	622	8	antennal esterase CXE20	*Spodoptera littoralis*	ACV60247	343	2E-112	81%

*Gene names as deposited in GenBank. ^§^Genbank accession number of the homologous gene. *CXE* carboxylesterase.

**Table 5:** **Candidate olfactory genes involved in *A. ipsilon *pheromone reception**

Gene Name*	**Unigene**	**Accession num.**	**Length (AA)**	**Signal peptide**	**No. of reads**	**Putative identification**	**Species**	**Accession num.**^**§**^	**Score (bits)**	***E*-value**	**% Identity**
**Chemosensory proteins**											
AipsCSP1	Unigene_468	JX863696	124	1-16 aa	21	chemosensory protein	*Heliothis virescens*	ACX53825	130	5E-36	59%
AipsCSP2	Unigene_1704	JX863697	119	1-16 aa	117	chemosensory protein	*Papili oxuthus*	BAF91716	159	8E-48	66%
AipsCSP3	Unigene_1767	JX863698	128	1-18 aa	19	chemosensory protein	*Mamestra brassicae*	AAF71290	223	2E-72	83%
AipsCSP4	Unigene_2047	JX863699	120	1-16 aa	46	chemosensory protein 2	*Helicoverpa armigera*	AEX07265	222	2E-72	86%
AipsCSP5	Unigene_6052	JX863700	107	1-18 aa	16	chemosensory protein	*Danaus plexippus*	EHJ67380	186	3E-55	84%
AipsCSP6	Unigene_15000	JX863701	127	1-18 aa	13	chemosensory protein 2	*Heliothis virescens*	AAM77040	227	3E-74	87%
AipsCSP7	Unigene_15308	JX863702	128	1-16 aa	18	chemosensory protein	*Heliothis virescens*	ACX53804	211	5E-68	75%
AipsCSP8	Unigene_721	JX863703	123	1-18 aa	390	chemosensory protein	*Heliothis virescens*	ACX53806	206	3E-66	76%
**Odorant binding proteins**											
AipsOBP1	Unigene_520	JX863689	183	ND	44	odorant binding protein	*Heliothis virescens*	ACX53761	197	2E-60	52%
AipsOBP2	Unigene_2120	JX863690	148	1-21 aa	44	pheromone binding protein 4	*Mamestra brassicae*	AAL66739	241	6E-77	79%
AipsOBP3	Unigene_6517	JX863691	108	ND	10	odorant-binding protein 19	*Helicoverpa armigera*	AFM93773	127	4E-34	53%
AipsOBP4	Unigene_8860	JX863692	121	ND	4	antennal binding protein	*Heliothis virescens*	CAC33574	173	7E-53	65%
AipsOBP5	Unigene_15218	JX863693	137	1-16 aa	20	odorant binding protein	*Heliothis virescens*	ACX53795	193	2E-60	70%
AipsOBP6	Unigene_15711	JX863694	125	ND	5	odorant binding protein	*Heliothis virescens*	ACX53743	243	7E-80	86%
AipsOBP7	Unigene_15861	JX863695	145	1-23 aa	4	odorant binding protein 3	*Helicoverpa armigera*	AEB54582	97.8	9E-23	38%

*Gene names as deposited in GenBank. ^§^Genbank accession number of the homologous gene. ND, not detected signal peptide because the N-terminus is missing. *CSP* chemosensory protein, *OBP* odorant binding protein.

#### Receptor for the pheromone biosynthesis activating neuropeptide (PBAN)

PBAN is released from the suboesophagal ganglion in the brain and goes to the hemolymph, where it binds to the PBAN receptor in the membrane of the pheromone gland and triggers the pheromone production [42,43]. Although there was no PBAN receptor found in the pheromone gland transcriptome of *H. virescens*[39] we found one transcript (*Unigene_3821*) encoding a protein highly homologous to PBAN receptor isoform B. It has very low abundance in the *A. ipsilon* transcriptome (31 RPKM) but high amino acid identity of 97% to *H. virescens* PBAN receptor in GenBank (Protein IDs: ABU93813) [44].

#### Acetyl-CoA carboxylase (ACC)

**Figure 5:**
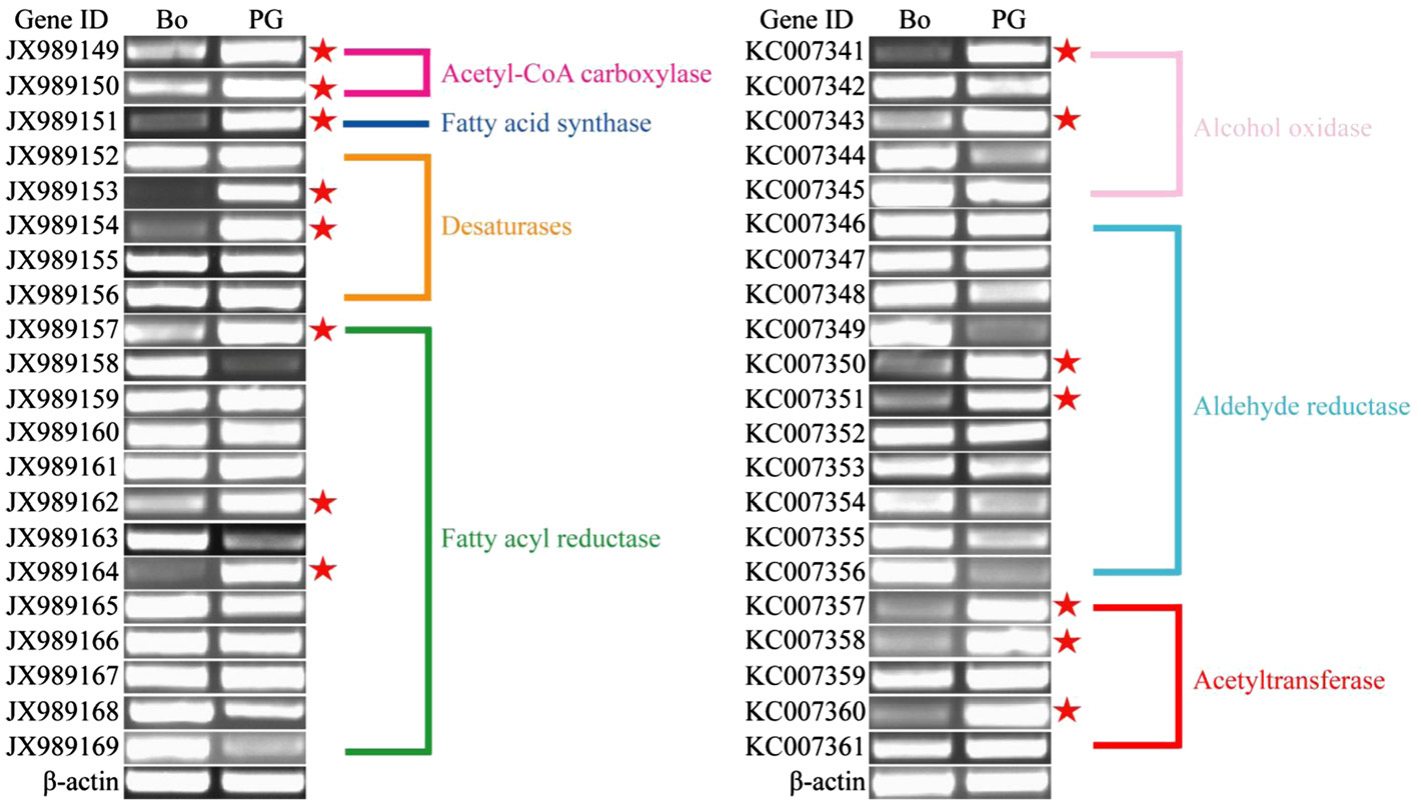
**RT-PCR results showing the relative expression of the *A. ipsilon *pheromone biosynthesis-related genes in pheromone gland (PG) and the body (BO).** The genes that are more highly expressed in the pheromone gland are labeled with red pentagram. *β-actin* was used as internal reference gene to test the integrity of each cDNA templates; the similar intensity of *β-actin* bands between the pheromone gland and the body part indicate the use of equal template concentrations.

**Figure 6:**
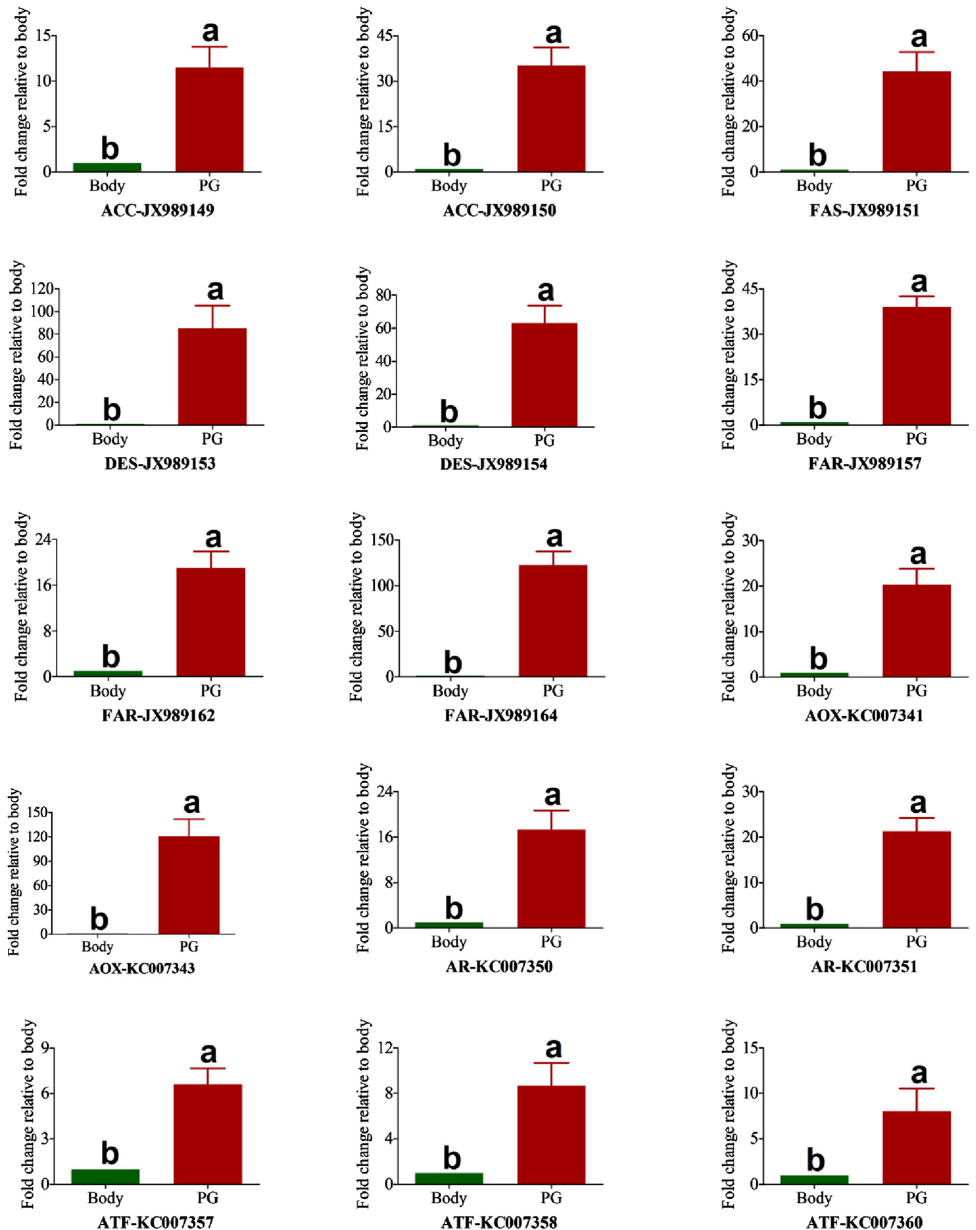
**qRT-PCR results showing the relative expression levels of the *A. ipsilon *pheromone biosynthesis related genes between the pheromone gland (PG) and the body (BO).** The putative enzyme names are indicated as gene abbreviations followed by Genbank accession numbers. *ACC* Acetyl-CoA carboxylase, *FAS* Fatty acid synthase, *DES* Desaturase, *FAR* Fatty acyl reductase, *AOX* alcohol oxidase, *AR* Aldehyde reductase, *ATF* Acetyltransferase. The internal control *β-actin* and *ribosomal protein S3* were used to normalize transcript levels in each sample. This figure was presented using *β-actin* as reference gene to normalize the target gene expression and correct sample-to-sample variation; similar results were also obtained with *ribosomal protein S3* as reference gene. The standard error is represented by the error bar, and the different letters (a, b) above each bar denote significant differences (p >0.05).

Saturated long chain fatty acids are the precursors of sex pheromones in most moth species. Their biosynthesis is started by ACC catalysing the production of malonyl-CoA from acetyl-CoA in the first committed biosynthesis step [8,9]. In the *A. ipsilon* pheromone gland we found two transcripts (*ACC-JX989149* and *ACC-JX989150*) encoding ACCs. *ACC-JX989149* with an open reading frame (ORF) of 5841 bp encodes for a ACC with 67% amino acid identity with the ACC of *T. castaneum* (Protein ID: XP_969851) and *ACC-JX989150* encodes a protein with 56% amino acid identity with the ACC of *H. virescens* (Protein ID: ACX53705) (Table 3). The RT-PCR and qRT-PCR revealed that both *ACC-JX989149* and *ACC-JX989150* are highly expressed in the pheromone gland as compared to the body (Figure 5 and Figure 6). However, they have very low abundance (81 and 21 RPKM) in the transcriptome (Figure 3).

#### Fatty acid synthase (FAS)

FAS has been shown to catalyse the conversion of malonyl-CoA and NADPH to produce saturated fatty acids [8]. We identified one putative *FAS* transcript (*FAS-JX989151*) in the *A. ipsilon* pheromone gland (Table 3), containing an ORF of 7176 bp and encoding a FAS with 57% amino acid identity to the FAS of *T. castaneum* (Protein ID: XP_970417). The RT-PCR and qRT-PCR revealed that *FAS-JX989151* is highly expressed in the pheromone gland (40-fold higher than in the body, Figure 5 and Figure 6) and also has a high abundance (343 RPKM) in the transcriptome (Figure 3).

#### Desaturase (DES)

**Figure 7:**
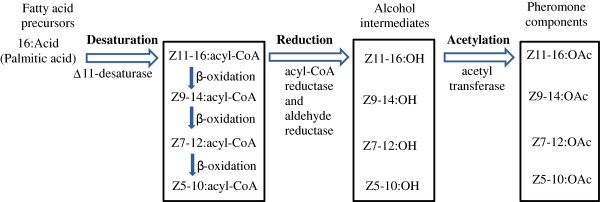
**Putative biosynthesis pathways of the sex pheromones in *Agrotis ipsilon*.** The saturated fatty acid precursor palmitic acid (16:0) is desaturated by ∆11-desaturase to form the precursor *Z*11-16:acyl-CoA for the production of three major and one minor pheromone components (adapted from [2,6,12,13,50]).

Pheromone-specific desaturases introduce double bond(s) into the fatty acids at specific positions along the chain. Five putative sex pheromone components extracted from *A. ipsilon* sex pheromone gland are unsaturated fatty acids with acetate as the functional group and 16 or less carbons [38]. At least three active pheromone components (*Z*7-12:OAc, *Z*9-14:OAc and *Z*11-16:OAc) have been identified in *A. ipsilon* strains from China [38], North America [45], France [46] and Japan [47]. It is reasonable to propose that the saturated fatty acid precursor of *A. ipsilon* sex pheromones would be palmitic acid (16:0) which is desaturated by ∆11-desaturase to form the precursor *Z*11-16:acyl-CoA for the production of two major (*Z*7-12:OAc and *Z*9-14:OAc) and two minor (*Z*11-16:OAc and Z5-10:Ac) pheromone components (Figure 7). It is not clear how the minor pheromone component (*Z*8-12:OAc) is synthesized in *A. ipsilon*, which should involve a ∆12-desaturase. Other studies in Lepidoptera species support a ∆11-desaturase acting on palmitic acid and leading to the production of the sex pheromone components [19,20,48]. In the *A. ipsilon* pheromone gland transctiptome 5 transcripts have high homology to genes encoding desaturases (Table 3). *DES-JX989152* is homologous to a gene encoding an acyl-CoA ∆9-desaturase in *M. brassicae* (Protein ID: ABX90048) with an amino acid identity of 96%. ∆9-desaturase makes oleic acid from stearic acid (18:0) and possibly palmitoleic acid from palmitic acid [16,17,49]. It would not participate in the biosynthesis of *A. ipsilon* sex pheromones. *DES-JX989153* encodes a protein with 87% amino acid identity with the acyl-CoA ∆11 desaturase of *M. brassicae* (Protein ID: ABX90049). *DES-JX989154*, *DES-JX989155* and *DES-JX989156* encode proteins, respectively, with 94% amino acid identity to the acyl-CoA desaturase from *H. assulta* (Protein ID: AF482909), 64% amino acid identity to a *S. littoralis* desaturase (Protein ID: AAQ74260) and 93% amino acid identity to an acyl-CoA desaturase of *S. exigua* (Protein ID: AAM28510). These transcripts could possibly encode ∆12-desaturases in *A. ipsilon* in formation of the minor pheromone component *Z*8-12:OAc from the precursor *Z*12-16:acyl-CoA. However, they could also function as ∆9-desaturase. Further study on their enzyme activity could confirm their role in the sex pheromone biosynthesis. The RT-PCR and qRT-PCR results indicated that *DES-JX989153* and *DES-JX989154* are highly expressed in the *A. ipsilon* pheromone gland compared with the body (85 and 63 fold higher, respectively) (Figure 5 and Figure 6). One of the transcripts (*DES-JX989154*) is also highly abundant (1206 RPKM) in the pheromone gland transcriptome (Figure 3), suggesting a possible role in *A. ipsilon* sex pheromone biosynthesis.

##### Fatty acyl-CoA reductase (FAR)

Once a specific Δ11 and possibly Δ12 double bond is introduced into fatty acid precursors to form a fatty acyl-CoA precursor, the chain of the precursors is then shortened sequentially by ß–oxidation to form different shorter chain fatty acyl-CoA precursors [6]. These precursors are further reduced individually by fatty acyl reductase (FAR) to form corresponding fatty alcohols [26,28,51]. In the *A. ipsilon* pheromone gland transcriptome there are 13 transcripts homologous to putative FAR genes (Table 3). Among them, 5 transcripts encode proteins with 59%-80% amino acid identity to the fatty-acyl CoA reductases of *Ostrinia nubilalis* (Protein IDs: ADI82776, ADI82777, ADI82778 and ADI82779). Other FAR transcripts are homologous to the fatty acyl-CoA reductase from a wide range of insect species including *H. virescens*, *N. vitripennis*, *Danaus plexippus*, *Bombus terrestris* and *Apis mellifera* with amino acid identities of about 60% (Table 3). The RT-PCR and qRT-PCR results indicated that three transcripts (*FAR-JX989157*, *FAR-JX989162* and *FAR-JX989164*) are highly expressed in the pheromone gland (Figure 5 and Figure 6). The other ten transcripts seem equally expressed in the pheromone gland and the body or highly expressed in the body. All FAR transcripts except two (*FAR-JX989157* and *FAR-JX989159*) have low abundance (from 81 and 16 RPKM) in the pheromone gland transcriptome (Figure 3).

##### Alcohol oxidase/dehydrogenase (*AOX*)

Fatty alcohols can be used as pheromone components in many moth species, and they are also pheromone intermediates to produce aldehyde pheromones by the alcohol oxidases [52,53]. In the *A. ipsilon* PG 5 homologous genes of alcohol oxidase/dehydrogenase were identified, the BLASTx results revealed three unigenes (*AOX-KC007341*, *AOX-KC007342* and *AOX-KC007344*) are with the amino acid identity of 43%, 55% and 64%, respectively, to a putative alcohol dehydrogenase of *D. plexippus* (Protein ID: EHJ70611), and one unigene (*AOX-KC007345*) are homologous to another putative alcohol dehydrogenase of *D. plexippus* (Protein ID: EHJ73729 ) with the amino acid identity of 68%. *AOX-KC007343* showed 78% amino acid identity with the alcohol dehydrogenase of *H. virescens* (Protein ID: ACX53694). The RT-PCR and qRT-PCR results indicated that *AOX-KC007341* and *AOX-KC007343* showed a higher expressed level in the PG than in the body (Figure 5 and Figure 6).

##### Aldehyde reductase (AR)

Aldehyde reductases are members of the aldo-ketoreductase superfamily and could be used to reduce long-chain acyl-CoA to form alcohol intermediates [13]. In the *A. ipsilon* pheromone gland we identified 11 transcripts with homology to the aldo-ketoreductases of *Papilio dardanus*, *B. mori*, *H. armigera*, *D. plexippus*, *Culex quinquefasciatus*, *H. virescens* and *Papilio xuthus* (Table 3). The derived protein sequences of these 11 transcripts show 53%-88% amino acid identity with their homologs in other insects. The RT-PCR and qRT-PCR results indicated that *AR-KC007350* and *AR-KC007351* are mainly expressed in the pheromone gland, while the other 9 putative aldehyde reductase transcripts have equal expression levels between the pheromone gland and the body or a higher expression level in the body (Figure 5 and Figure 6). All aldehyde reductase transcripts are present at low abundance (from 67 to 10 RPKM) in the pheromone gland transcriptome (Figure 3). The involvement of aldehyde reductase in sex pheromone biosynthesis has not been demonstrated in moth species.

##### Acetyltransferase (ATF)

The fatty acid alcohols are used as pheromone components in many moth species. In *A. ipsilon* whose sex pheromone blends comprise only acetates, they are intermediates and acetylated to pheromone components as acetate esters by actyltransferases [13]. In the *A. ipsilon* pheromone gland transcriptome 5 acetyltransferase homologous transcripts were identified (Table 3), 3 of them (*ATF-KC007357*, *ATF-KC007360* and *ATF-KC007361*) encode proteins that are homologous to the acetyltransferase of *D. plexippus* (Protein IDs: EHJ65205, EHJ65977 and EHJ68573) with relatively high amino acid identities (<70%), one (*ATF-KC007358)* encodes a protein with 90% amino acid identity to *H. virescens* acetyltransferase (Protein ID: ACX53812) and one (*ATF-KC007359*) encodes a protein with 86% amino acid identity with the acetyltransferase of *B. mori* (Protein ID: NP_001182381). The RT-PCR and qRT-PCR revealed that three transcripts (*ATF-KC007358*, *ATF-KC007360* and *ATF-KC007357*) are mainly expressed in the pheromone gland (Figure 5 and Figure 6) and have a relative high abundance of 195, 155 and 71 RPKM, respectively in the pheromone gland transcriptome (Figure 3).

#### Genes encoding candidate pheromone degrading enzymes in the *A. ipsilon* pheromone gland

**Figure 8:**
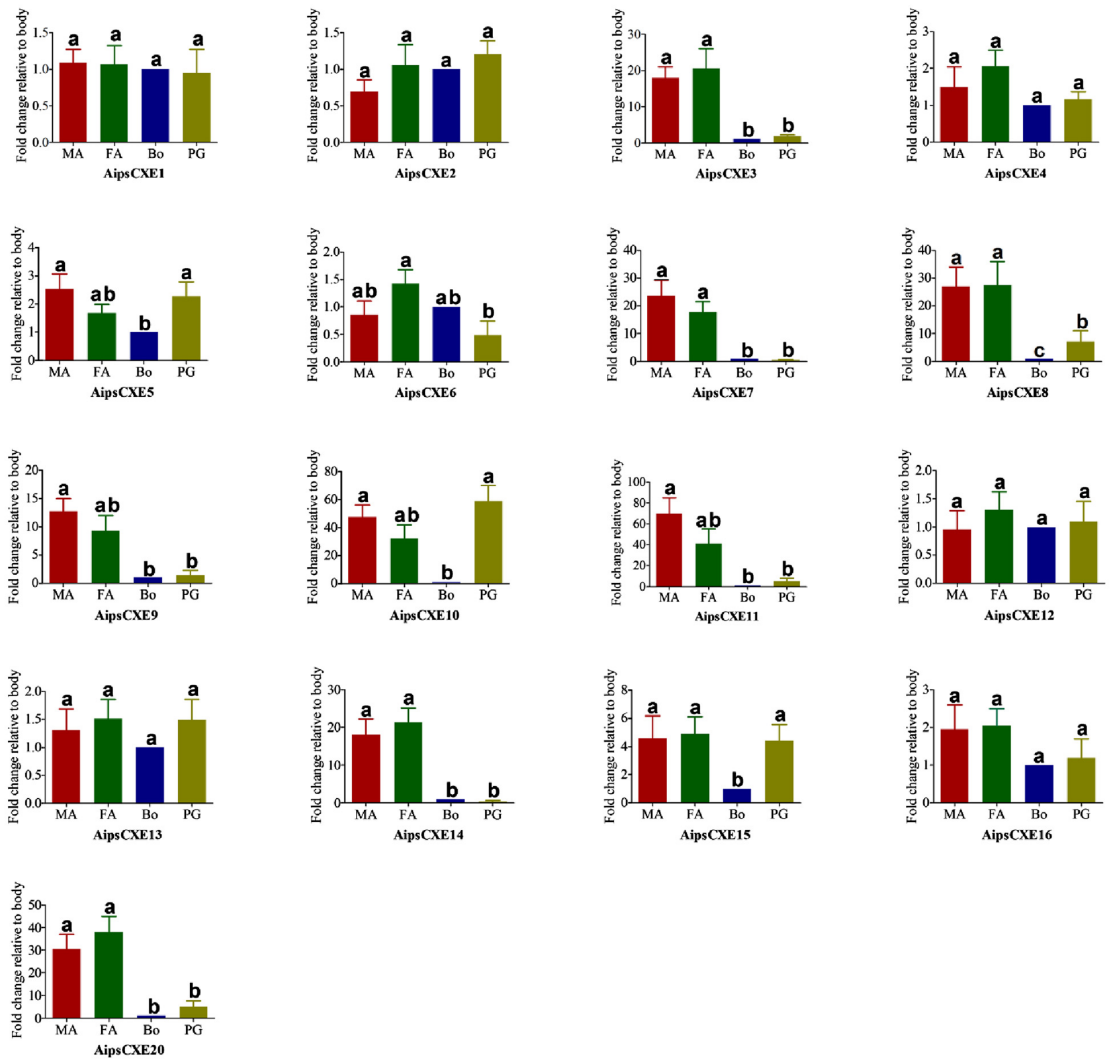
**qRT-PCR results showing the expression of *A. ipsilon *unigenes encoding the putative esterase (CXE) identified in the pheromone gland in the male antennae (MA), the female antennae (FA), the body (BO) and the pheromone gland (PG).** The standard error is represented by the error bar, and the different letters (a, b, c) above each bar denote significant differences (p > 0.05).

It would be potentially harmful to insects if pheromone molecules and other odorants remained on the olfactory receptors after they had stimulated the olfactory receptor neurons (ORNs). It is therefore thought that there are mechanisms to protect the ORNs by odorant degrading enzymes (ODEs) [37] including esterases [54,55], aldehyde oxidases [56-58], cytochromes P450 [59-61], carboxyl esterase [62], and glutathione S-transferase (GST) [63]. In this study, we identified 17 transcripts predicted to encode esterases in the *A. ipsilon* pheromone gland, and the BLASTx results showed that all have very high amino acid identities with the antennal esterases of *S. littoralis* (Table 4), we named them as *AipsCXE1-AipsCXE16* and *AipsCXE20* following the nomenclature in *S. littoralis.* Our qRT-PCR results revealed that 7 of the transcripts (*AipsCXE3*, *AipsCXE7*, *AipsCXE8*, *AipsCXE9*, *AipsCXE11*, *AipsCXE14* and *AipsCXE20*) are antennal-enriched, 3 (*AipsCXE5*, *AipsCXE10* and *AipsCXE15*) are both antennal- and pheromone gland-enriched and the remaining 7 (*AipsCXE1*, *AipsCXE2*, *AipsCXE4*, *AipsCXE6*, *AipsCXE12*, *AipsCXE13* and *AipsCXE16*) have similar expression levels in antennae, body and pheromone gland, suggesting they are not pheromone specific (Figure 8).

#### Genes encoding candidate pheromone carrier proteins in the *A. ipsilon* pheromone gland

**Figure 9:**
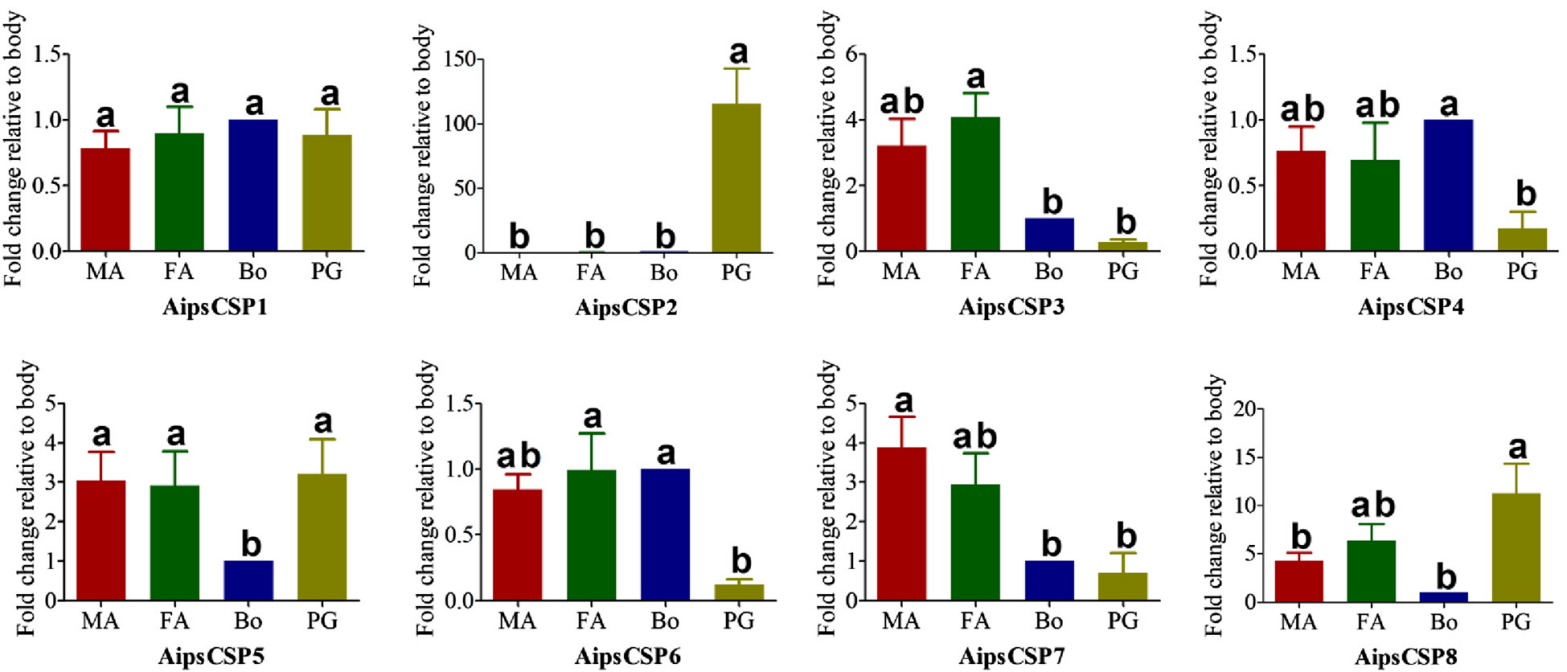
**qRT-PCR results showing the relative expression of the *A. ipsilon *unigenes encoding putative chemosensory proteins (CSP) identified in the pheromone gland in the male antennae (MA), the female antennae (FA), the body (BO) and the pheromone gland (PG).** The standard error is represented by the error bar, and the different letters (a, b) above each bar denote significant differences (p >0.05).

Moth sex pheromones are synthesised and protected from degradation until being released from the female pheromone gland and it has been proposed that OBPs and CSPs could participate in this process. In this study we have identified transcripts of 7 OBPs and 8 CSPs from the *A. ipsilon* pheromone gland (Table 5), all of these have the typical insect OBP sequence motif C1-X_15-39_-C2-X_3_-C3-X_21-44_-C4-X_7-12_-C5-X_8_-C6 [35,64] or CSP sequence motif C_1_-X_6-8_-C_2_-X_16-21_-C_3_-X_2_-C_4_[65]. One CSP transcript, *AipsCSP2* seems to be gland-specific and has an extremely high expression level (<100 folds) in the pheromone glands compared with the antennae and body and a relative high abundance in the pheromone gland transcriptome. *AipsCSP8* shows a higher expression level in the pheromone gland (10-fold higher than in body) (Figure 9) and is extremely abundant with 1,364 RPKM in the pheromone gland transcriptome (Figure 4).

**Figure 10:**
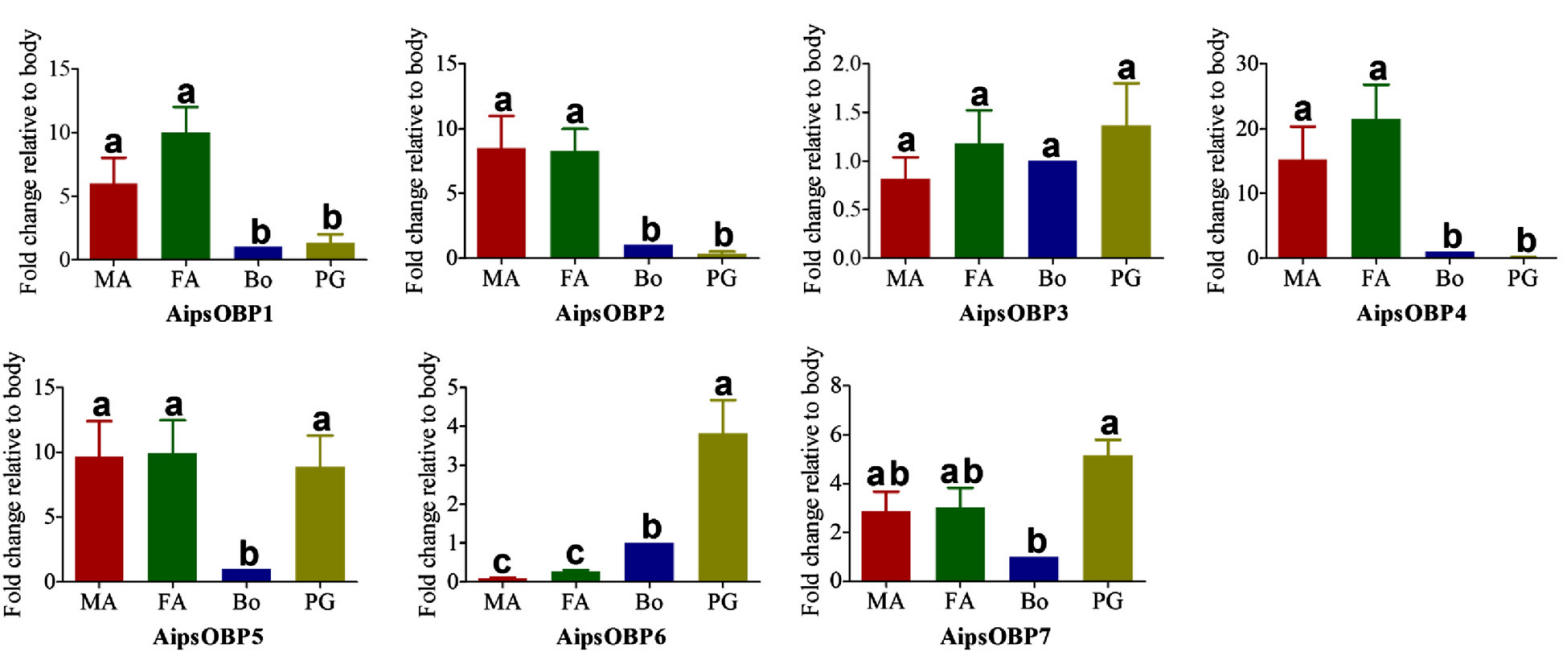
**qRT-PCR results showing the relative expression of the *A. ipsilon *unigenes encoding putative odorant binding proteins (OBP) identified in the pheromone gland in the male antennae (MA), the female antennae (FA), the body (BO) and the pheromone gland (PG).** The standard error is represented by the error bar, and the different letters (a, b, c) above each bar denote significant differences (p > 0.05).

There is one OBP transcript (*AipsOBP6*) which is highly expressed in the pheromone gland (more than 3-fold higher than in the antennae), and 3 OBPs (*AipsOBP1, AipsOBP2* and *AipsOBP4*) are highly expressed in the antennae (Figure 10). This high expression of OBPs and CSPs in the pheromone gland is interesting because it suggests a possible involvement in carrying and releasing sex pheromones as demonstrated for the antennal OBPs and CSPs. However, the molecular mechanisms that connect these proteins with the involvement of pheromone production needs further investigation. No ORs, IRs and SNMPs are identified in the *A. ipsilon* pheromone gland.

## Conclusions

The black cutworm *A. ipsilon* is a destructive pest of many crops [66,67] and mainly controlled by chemical pesticides, which has led to the development of resistance to various compounds [68]. Our study provides information and resource to identify and facilitate functional studies of genes responsible for pheromone production, transport and degradation at the molecular level both *in vivo* and *in vitro*.

By deep sequencing of the *A. ipsilon* sex pheromone gland transcriptome, we have identified 42 transcripts encoding enzymes putative involved in pheromone production. This is the first study reporting the key enzyme ∆11-desaturase involved in *A. ipsilon* sex pheromone biosynthesis. One new transcript (*DES-JX989154*) encoding a desaturase is highly abundant in the transcriptome and highly expressed in the pheromone gland, suggesting this desaturase encoded by *DES-JX989154* or other newly identified transcripts (*DES-JX989155* and *DES-JX989156*) may play important roles in *A. ipsilon* sex pheromone biosynthesis. They may contribute in the introducing a double bond at C11 and C12 positions of the saturated fatty acid precursor palmitic acid for the production of pheromone precursors. Further studies are needed to confirm the substrates and the products thus the involvement of these desaturases and other newly identified genes such as those encoding for aldehyde reductases and acetyltransferases in *A. ipsilon* sex pheromone biosynthesis. Two of the CSPs are highly abundant transcripts (*AipsCSP2* and *AipsCSP8*) with 100- and 10-fold higher transcription level, respectively than in the body. Furthermore *AipsCSP2* and *AipsOBP6* are pheromone gland-specific and –enriched, respectively (Figure 9 and Figure 10). This suggests a functional role of the PG-enriched CSPs and OBPs in sex pheromone transport and release. It is clear that during perireceptor event after pheromones and odorants enter the sensillun lymph that the antennae-specific odorant binding proteins (OBPs) capture these hydrophobic pheromone and odorant and deliver them to the membrane-bound olfactory receptors (ORs) [35]. Further study of these PG-expressed OBPs, especially their binding to sex pheromone components is needed to confirm its function.

## Methods

### Insect material

The *A. ipsilon* colony has been reared in our laboratory (State Key Laboratory for Biology of Plant Diseases and Insect Pests, Chinese Academy of Agricultural Sciences, Beijing, China) since 2006 with field-collected moths introduced each summer to prevent inbreeding effects. The larvae were reared on an artificial diet comprising wheat germ, casein and sucrose as the main components. The colony was kept at 24°C with 75% relative humidity and a 14h:10h light:dark photoperiod. Pupae were sexed and kept separately in hyaline plastic cups before emergence. Adult moths were given 20% honey solution after emergence.

### Pheromone gland dissection

The pheromone gland plus associated ovipositor valves and parts of the terminal abdominal segments were dissected with fine scissors [39] from the rest of the body parts refereed as ‘body’ which comprises of heads, thoraxes, legs, wings and abdomens (without the pheromone glands). The calling behavior of female *A. ipsilon* moths begins on the first night after eclosion and increases sharply, peaking on the third night [38]. So in order to cover all genes involved in pheromone biosynthesis, four glands of 1-day-old females, four glands of 2-day-old females and ten glands of 3-day-old females were dissected during the second half of the scotophase, which is reported to be the calling period of this moth [69-71]. The eighteen glands were mixed in one RNase-free centrifuge tube for total RNA extraction and frozen in liquid nitrogen until further processing.

### RNA extraction and cDNA library construction

Total RNA was extracted using TRIzol regent (Invitrogen, Carlsbad, CA, USA) according to the manufacturer’s protocol. The quantity of RNA was determined using a Nanodrop spectrophotometer (Thermo Scientific, Wilmington, DE, USA) and 1.1% agarose gel electrophoresis. About 500 ng mRNA was further purified from 50 μg total RNA using the polyATtract mRNA isolation system III (Promega, Madison, WI, USA). The mRNA was then sheared into about 800 nucleotides using a RNA fragmentation solution (Autolab, Beijing, China) at 70°C for 30 sec, and then cleaned and condensed using RNeasyMinElute RNA Cleaning Up kit (Qiagen, Valencia, CA, USA). The mRNA was used as a template for first-strand cDNA synthesis using N6 random primers and MMLV reverse transcriptase (TaKaRa, Dalian, China) and the second strands were synthesized using Secondary Strand cDNA synthesis enzyme mixtures (Autolab, Beijing, China). cDNAs with appropriate length were purified with the QIAquick PCR Purification kit (Qiagen, Valencia, CA, USA) and eluted with 10 μl Elution Buffer. After blunt ending and the addition of a poly-A tail at the 3’ end according to the Roche’s Rapid Library Preparing protocols (Roche, USA), the purified cDNAs were linked to GS-FLX sequencing Adaptors (Roche, USA). Finally, the cDNAs shorter than 500 bp were removed using Ampure Beads according to the manufactures’ instruction (Beckman, USA) before the preparation of the cDNA library.

### 454 sequencing

Pyrosequencing of the cDNA library was performed by Beijing Autolab Biotechnology Company using a 454 GS-FLX sequencer (Roche, IN, USA). All sequencing reads were deposited into the Short Read Archive (SRA) of the National Center for Biotechnology Information (NCBI) under the accession number SRX189143.

### Sequence analysis and assembly

Base calling of the raw 454 reads in SFF files was carried out using the python script sff_extract.py developed by COMAV (http://bioinf.comav.upv.es). All raw reads were then processed to remove low quality and adaptor sequences using programs tagdust [72], LUCY [73] and SeqClean [74] with default parameters. The resulting sequences were then screened against the NCBI UniVec database (http://www.ncbi.nlm.nih.gov/VecScreen/UniVec.html) to remove possible vector sequence contamination. Cleaned reads shorter than 60 bases were discarded because they are likely to be sequencing artifacts [75].

Two steps were taken to assemble the clean reads. First MIRA3 [76] was used with the assembly settings of minimum sequence overlap of 30 bp and minimum percentage overlap identity of 80%. Then CAP3 was used with assembly parameters of overlap length cutoff <30 and overlap percent identity cutoff <90% [77]. The resulting contigs and singletons of more than 100 bases were retained as unigenes and annotated as described below.

### Homology searches and functional classification

Following the assembly, homology searches of all unigenes were performed using BLASTx and BLASTn programs against the GenBank non-redundant protein (nr) and nucleotide sequence (nt) database at NCBI [78]. Matches with an E-value less than 1.0E-5 were considered significant [79]. Gene names were assigned to each unigene based on the best BLASTx hit with the highest score value.

Gene Ontology terms were assigned by Blast2GO [80] through BLASTx program with an E-value less than 1.0E-5. Then, WEGO [81] software was used for assignment of each GO ID to the related ontology entries. The longest open reading frame (ORF) of each unigene was determined by an ORF finder tool (http://www.ncbi.nlm.nih.gov/gorf/gorf.html).

### Pheromone gland ESTs from other insects

The *H. virescens* pheromone gland ESTs (14112 with accession number: GR958232-GR972305, GT067784-GT067747) [39], the *A. segetum* pheromone gland ESTs (2286 with accession number: ES582156-ES584441) [82] and the *B. mori* pheromone gland ESTs (10501 with accession number: BP184340-BP182009; AV404455-AV403746; DC552314-DC544856) were downloaded from the dbEST database at NCBI (http://www.ncbi.nlm.nih.gov/nucest) and saved as fasta files. All the EST sequences were assembled using the CAP3 program with the same parameters as used in the *A. ipsilon* assembly. The comparative analyses of *A. ipsilon*, *H. virescens*, *B. mori* and *A. segetum* pheromone gland unigenes were performed based on the Best Bidirectional Hits results (reciprocal BLASTn, E-value less than 1.0E-6).

### Identification of candidate genes associated with moth pheromone biosynthesis

Some putative genes and enzymes have been reported previously as being involved in moth sex pheromone production. We focused our research on the target genes: (1) Acetyl-CoA carboxylase; (2) Fatty acid synthase; (3) Desaturase; (4) Fatty acyl reductase; (5) Alcohol oxidase; (6) Aldehyde reductase; (7) Acetyltransferase.

### Identification of putative genes involved in pheromone degradation

Since the sex pheromone blend of *A. Ipsilon* is comprised of acetate esters (*Z*)-7-dodecenyl acetate (Z7-12:Ac) (40.5%), (*Z*)-9-tetradecenyl acetate (Z9-14:Ac) (13.2%), (*Z*)-11-hexadecenyl acetate (Z11-16:Ac) (14.9%), (*Z*)-8-dodecenyl acetate (Z8-12:Ac) (17.2%) and (*Z*)-5-decenyl acetate (Z5-10:Ac) (14.3%) [38], esterases may play a major role in pheromone degradation. Therefore, we performed BLASTx and BLASTn searches to identify candidate esterase genes in the *A. ipsilon* pheromone gland NGS dataset.

### Identification of putative genes involved in pheromone transport

Genes encoding odorant binding proteins (OBPs) and chemosensory proteins (CSPs) were identified using the “OBP sequence motif” C1-X_15-39_-C2-X_3_-C3-X_21-44_-C4-X_7-12_-C5-X_8_-C6 [64] and the “CSP sequence motif” C_1_-X_6-8_-C_2_-X_16-21_-C_3_-X_2_-C_4_, [65]. Candidate olfactory receptors (ORs), ionotropic receptors (IRs), sensory neuron membrane proteins (SNMPs) genes were identified by BLASTx and BLASTn searches.

### Sequence analyses

The putative N-terminal signal peptides and most likely cleavage sites were predicted by the SignalP V3.0 program [83] (http://www.cbs.dtu.dk/services/SignalP/). Sequence alignments were done with ClustalX 1.83 [84] with default gap penalty parameters of gap opening 10 and extension 0.2.

### RT-PCR and qRT-PCR

The cDNAs from female pheromone glands and other body parts (mixture of heads, thoraxes, legs, wings and abdomens (without the pheromone glands)) were synthesized using PrimeScript RT Reagent with gDNA Eraser (TaKaRa, Dalian, China). 200 ng cDNA was used as RT-PCR and qRT-PCR templates. Specific primer pairs for RT-PCR analysis were designed with Primer 3 (http://frodo.wi.mit.edu/) or Primer Premier 5 (see Additional file 4). To test the integrity of the cDNA templates, a pair of control primers for the β-actin (GenBank Acc. JQ822245) of *A. ipsilon* was used. The PCR cycling profile was: 95°C for 2 min, followed by 35 cycles of 95°C for 30 sec, 60°C for 30 sec, 72°C for 1 min and a final extension for 10 min at 72°C. PCR products were separated in 1.2% agarose gels and stained with ethidium bromide. Each reaction was done at least six times with three biological replicates.

qRT-PCR analysis was conducted using the ABI 7500 Real-Time PCR System (Applied Biosystems, Carlsbad, CA). The primers were designed by Beacon Designer 7.90 (PREMIER Biosoft International) (see Additional file 5). Two reference genes, *β-actin* (GenBank Acc. JQ822245) and *ribosomal protein S3* (GenBank Acc. JQ822246) were used for normalizing expression of the target gene and correcting for sample-to-sample variation. qRT-PCRs were done in a 25 μl reaction containing 12.5 μl of Platinum SYBR Green qPCR SuperMix-UDG (Invitrogen, Shanghai, China), 0.5 μl of each primer (10 pmol/ μl), 0.5 μl of Rox Reference Dye, 1 μl of sample cDNA (200 ng/μl), 10 μl of sterilized H_2_O. The cycling parameters were: 50°C for 2 min, 95°C for 2 min, followed by 40 cycles of 95°C for 15 sec and 60°C for 30 sec. Then, the PCR products were heated to 95°C for 15 sec, cooled to 60°C for 1 min and heated to 95°C for 30 sec and cooled to 60°C for 15 sec to measure the dissociation curves. Negative controls, without either template or transcriptase, were included in each experiment. To check reproducibility, each qRT-PCR reaction for each sample was carried out in three technical replicates and three biological replicates.

### qRT-PCR data analysis

Relative quantification was performed using the comparative 2^-ΔΔCt^ method [85]. All data were normalized to endogenous *β-actin* or *ribosomal protein S3* levels from the same individual samples. In the analysis of the relative fold change in different tissues, the body sample was taken as the calibrator. Thus, the relative fold change in different tissues was assessed by comparing the expression level of each target gene in other tissues to that in the body part. The results are presented as the mean of the fold change in three biological samples. The comparative analyses of each *OBP, CSP and CXE* gene among different tissues were determined with one-way nested analysis of variance (ANOVA), followed by a Tukey’s honestly significance difference (HSD) test using SPSS Statistics 18.0 (SPSS Inc., Chicago, IL, USA). The comparative analyses of each putative pheromone biosynthesis gene between pheromone gland (PG) and body part were determined with paired *t*-test. When applicable, values were presented as mean ± SE.

## Abbreviations

CSP: Chemosensory protein; OBP: Odorant binding protein; CXE: Carboxylesterase; EST: Expressed sequenced tag; OR: Olfactory receptor; IR: Ionotropic receptor; SNMP: Sensory neuron membrane protein; ODE: Odorant-degrading enzyme; NGS: Next generation sequencing; PCR: Polymerase chain reaction; PDE: Pheromone degrading enzyme; PBP: Pheromone binding protein; PBAN: Pheromone biosynthesis activating neuropeptide; ACC: Acetyl-CoA carboxylase; FAS: Fatty acid synthetase; DES: Desaturase; FAR: Fatty acyl-CoA reductase; AR: Aldehyde reductase; ATF: Acetyltransferase; AOX: Aldehyde oxidase; GST: Glutathione S-transferase; Z7-12:OAc: (Z)-7-dodecenyl acetate; Z9-14:OAc: (Z)-9-tetradecenyl acetate; Z11-16:OAc: (Z)-11-hexadecenyl acetate; Z5-10:OAc: (Z)-5-decenyl acetate; Z8-12:OAc: (Z)-8-dodecenyl acetate; ORN: Olfactory receptor neuron.

## Competing interests

The author(s) declare that they have no competing interests.

## Authors’ contributions

SHG, YJZ and JJZ initiated the project, conceived and design the study. SHG, YJZ and JJZ wrote the manuscript. SHG carried out sample collection, library construction, the data processing, bioinformatics analysis, RT-PCR and qRT-PCR. YYG and KMW coordinated the study. JAP and LMF contributed data analyses, data interpretation and extensively revised the manuscript. All authors have read and approved the final manuscript.

## Supplementary Material

Additional file 1 Size distribution of the clean reads (A) and the assembled unigenes (B). Click here for file

Additional file 2 **The identified transcripts with putative roles in *Agrotis ipsilon* sex pheromone biosynthesis and transport (Tables** 3**,**4**,**5**).** Click here for file

Additional file 3 **Gene Ontology (GO) classifications of the 23473 *A. ipsilon* pheromone gland unigenes according to their involvement in biological processes, cellular component and molecular function.** Click here for file

Additional file 4 **Primers used for RT-PCR analysis of enzyme genes of the *A. ipsilon* PG.** Click here for file

Additional file 5 Primers used in real-time PCR for determination expression level of target genes. Click here for file

## References

[B1] Ando T Inomata S Yamamoto M Lepidopteran sex pheromones Top Curr Chem 2004 239 51 96 10.1007/b95449 22160231

[B2] Tillman JA Seybold SJ Jurenka RA Blomquist GJ Insect pheromones—an overview of biosynthesis and endocrine regulation Insect Biochem Mol Biol 1999 29 481 514 10.1016/S0965-1748(99)00016-8 10406089

[B3] McNeil JN Behavioral ecology of pheromone-mediated communication in moth and its importance in the use of pheromone traps Annu Rev Entomol 1991 36 407 430 10.1146/annurev.en.36.010191.002203

[B4] Witzgall P Stelinski L Gut L Thomson D Codling moth management and chemical ecology Annu Rev Entomol 2008 53 503 522 10.1146/annurev.ento.53.103106.093323 17877451

[B5] Witzgall P Kirsch P Cork A Sex pheromones and their impact on pest management J Chem Ecol 2010 36 80 100 10.1007/s10886-009-9737-y 20108027

[B6] Jurenka R Insect pheromone biosynthesis Top Curr Chem 2004 239 97 132 10.1007/b95450 22160232

[B7] Matsumoto S Molecular mechanisms underlying sex pheromone production in moths Biosci Biotechnol Biochem 2010 74 223 231 10.1271/bbb.90756 20139627

[B8] Volpe JJ Vagelos PR Saturated fatty acid biosynthesis and its regulation Annu Rev Biochem 1973 42 21 60 10.1146/annurev.bi.42.070173.000321 4147183

[B9] Pape ME Lopez-Casillas F Kim KH Physiological regulation of acetyl-CoA carboxylase gene expression: effects of diet, diabetes, and lactation on acetyl-CoA carboxylase mRNA Arch Biochem Biophys 1988 267 104 109 10.1016/0003-9861(88)90013-6 2904242

[B10] Bjostad LB Roelofs WL Biosynthesis of sex pheromone components and glycerolipid precursors from sodium [1–14C] acetate in redbanded leafroller moth J Chem Ecol 1984 10 681 691 10.1007/BF00994228 24318604

[B11] Tang JD Charlton RE Jurenka RA Wolf WA Phelan PL Sreng L Roelofs WL Regulation of pheromone biosynthesis by a brain hormone in two moth species Proc Natl Acad Sci USA 1989 86 1806 1810 10.1073/pnas.86.6.1806 16594018 PMC286793

[B12] Jurenka RA Jacquin E Roelofs WL Control of the pheromone biosynthetic pathway in *Helicoverpa zea* by the pheromone biosynthesis activating neuropeptide Arch Insect Biochem Physiol 1991 17 81 91 10.1002/arch.940170203

[B13] Morse D Meighen EA Prestwich GD, Bloriiquist GJ Pheromone biosynthesis: enzymatic studies in lepidoptera Pheromone Biochemistry 1987 Orlando, FL: Academic Press 121 158

[B14] Hoskovec MA Luxová A Svatoš A Boland W Biosynthesis of sex pheromones in moths: stereochemistry of fatty alcohol oxidation in *Manduca sexta* Tetrahedron 2002 58 9193 9201 10.1016/S0040-4020(02)01199-7

[B15] Foster SP Roelofs WL Sex pheromone biosynthesis in the tortricid moth, *Ctenopseustis herana* (Felder & Rogenhofer) Arch Insect Biochem Physiol 1996 33 135 147 10.1002/(SICI)1520-6327(1996)33:2<135::AID-ARCH4>3.0.CO;2-X

[B16] Löfstedt C Bengtsson M Sex pheromone biosynthesis of (*E, E*)-8,10-dodecadienol in codling moth *Cydia pomonella* involves *E*9 desaturation J Chem Ecol 1988 14 903 915 10.1007/BF01018782 24276140

[B17] Martinez T Fabriás G Camps F Sex pheromone biosynthetic pathway in *Spodoptera littoralis* and its activation by a neurohormone J Biol Chem 1990 265 1381 1387 2295634

[B18] Foster SP Roelofs WL Sex pheromone biosynthesis in the leafroller moth *Planotortix excessana* by Δ10 desaturation Arch Insect Biochem Physiol 1988 8 1 9 10.1002/arch.940080102

[B19] Bjostad LB Roelofs WL Sex pheromone biosynthesis from radiolabeled fatty acids in the redbanded leafroller moth J Biol Chem 1981 256 7936 7940 7021542

[B20] Bjostad LB Roelofs WL Sex pheromone biosynthesis in *Trichoplusia ni*: key steps involve delta-11 desaturation and chain-shortening Science 1983 220 1387 1389 10.1126/science.220.4604.1387 17730655

[B21] Zhao C Löfstedt C Wang X Sex pheromone biosynthesis in the Asian corn borer *Ostrinia furnacalis* (II): biosynthesis of (*E*)- and (*Z*)-12-tetradecenyl acetate involves Δ14 desaturation Arch Biochem Physiol 1990 15 57 65 10.1002/arch.940150106

[B22] Lassance JM Liénard MA Antony B Qian S Fujii T Tabata J Ishikawa Y Löfstedt C Functional consequences of sequence variation in the pheromone biosynthetic gene pgFAR for *Ostrinia* moths Proc Natl Acad Sci USA 2013 110 3967 3972 10.1073/pnas.1208706110 23407169 PMC3593903

[B23] Teal PEA Tumlinson JH The role of alcohols in pheromone biosynthesis by 2 noctuid moths that use acetate pheromone components Arch Insect Biochem Physiol 1987 4 261 269 10.1002/arch.940040404

[B24] Roelofs WL Wolf WA Pheromone biosynthesis in Lepidoptera J Chem Ecol 1998 14 2019 2031 24277141 10.1007/BF01014247

[B25] Antony B Fujii T Moto K Matsumoto S Fukuzawa M Nakano R Tatsuki S Ishikawa Y Pheromone-gland-specific fatty-acyl reductase in the adzuki bean borer, *Ostrinia scapulalis* (Lepidoptera: Crambidae) Insect Biochem Mol Biol 2009 39 90 95 10.1016/j.ibmb.2008.10.008 19041942

[B26] Hagström AK Lienard MA Groot AT Hedenström E Löfstedt C Semi-selective fatty acyl reductases from four heliothine moths influence the specific pheromone composition PLoS ONE 2012 7 5 e37230 10.1371/journal.pone.0037230 22615947 PMC3353883

[B27] Lassance JM Groot AT Liénard MA Antony B Borgwardt C Andersson F Hedenström E Heckel DG Löfstedt C Allelic variation in a fatty-acyl reductase gene causes divergence in moth sex pheromones Nature 2010 466 486 489 10.1038/nature09058 20592730

[B28] Lienard MA Hagström AK Lassance JM Löfstedt C Evolution of multicomponent pheromone signals in small ermine moths involves a single fatty-acyl reductase gene Proc Natl Acad Sci USA 2010 107 10955 10960 10.1073/pnas.1000823107 20534481 PMC2890718

[B29] Moto K Kojima H Kurihara M Iwami M Matsumoto S Cell-specific expression of enhanced green fluorescence protein under the control of neuropeptide gene promoters in the brain of the silkworm, *Bombyx mori*, using *Bombyx mori* nucleopolyhedrovirus-derived vectors Insect Biochem Mol Biol 2003 33 7 12 10.1016/S0965-1748(02)00185-6 12459195

[B30] Moto K Suzuki MG Hull JJ Kurata R Takahashi S Yamamoto M Okano K Imai K Ando T Matsumoto S Involvement of a bifunctional fatty-acyl desaturase in the biosynthesis of the silkmoth, *Bombyx mori*, sex pheromone Proc Natl Acad Sci USA 2004 101 8631 8636 10.1073/pnas.0402056101 15173596 PMC423246

[B31] Ohnishi A Hull JJ Matsumoto S Targeted disruption of genes in the *Bombyx mori* sex pheromone biosynthetic pathway Proc Natl Acad Sci USA 2006 103 4398 4403 10.1073/pnas.0511270103 16537410 PMC1450183

[B32] Honson NS Gong Y Plettner E Structure and function of insect odorant and pheromone-binding proteins (OBPs and PBPs) and chemosensory-specific proteins (CSPs) Recent Ad Phytochem 2005 39 227 268

[B33] Pelosi P Zhou JJ Ban LP Calvello M Soluble proteins in insect chemical communication Cell Mol Life Sci 2006 63 1658 1676 10.1007/s00018-005-5607-0 16786224 PMC11136032

[B34] Leal WS Odorant reception in insects: roles of receptors, binding proteins, and degrading enzymes Annu Rev Entomol 2013 58 373 391 10.1146/annurev-ento-120811-153635 23020622

[B35] Zhou JJ Odorant-binding proteins in insects Vitam Horm 2010 83 241 272 20831949 10.1016/S0083-6729(10)83010-9

[B36] Vogt RG Riddiford LM Scale esterase a pheromone degrading enzyme from the wing scales of the silk moth *Antheraea polyphemus* J Chem Ecol 1986 12 469 482 10.1007/BF01020567 24306791

[B37] Prestwich GD Chemistry of pheromone and hormone metabolism in insects Science 1987 237 999 1006 10.1126/science.3616631 3616631

[B38] Xiang YY Yang MF Li ZZ Sex pheromone components of the female black cutworm moth in China: identification and field trials Zool Res 2009 30 59 64 10.3724/SP.J.1141.2009.01059

[B39] Vogel H Heidel AJ Heckel DG Groot AT Transcriptome analysis of the sex pheromone gland of the noctuid moth *Heliothis virescens* BMC Genomics 2010 11 29 10.1186/1471-2164-11-29 20074338 PMC2820457

[B40] Engelmann F Insect vitellogenin: identification, biosynthesis, and role in vitellogenesis Adv Insect Physiol 1979 14 49 108

[B41] Du M Yin X Zhang S Zhu B Song Q An S Identification of lipases involved in PBAN stimulated pheromone production in *Bombyx mori* using the DGE and RNAi approaches PLoS ONE 2012 7 e31045 10.1371/journal.pone.0031045 22359564 PMC3281041

[B42] Hull JJ Ohnishi A Moto K Kawasaki Y Kurata R Suzuki MG Matsumoto S Cloning and characterization of the pheromone biosynthesis activating neuropeptide receptor from the silkmoth, *Bombyx mori*: significance of the carboxyl terminus in receptor internalization J Biol Chem 2004 279 51500 51507 10.1074/jbc.M408142200 15358772

[B43] Rafaeli A Bober R Becker L Choi MY Fuerst EJ Jurenka R Spatial distribution and differential expression of the PBAN receptor in tissues of adult *Helicoverpa* spp. (Lepidoptera: Noctuidae) Insect Mol Biol 2007 16 287 293 10.1111/j.1365-2583.2007.00725.x 17328713

[B44] Kim YJ Nachman RJ Aimanova K Gill S Adams ME The pheromone biosynthesis activating neuropeptide (PBAN) receptor of *Heliothis virescens*: identification, functional expression, and structure-activity relationships of ligand analogs Peptides 2008 29 268 275 10.1016/j.peptides.2007.12.001 18243415 PMC3900413

[B45] Gemeno C Haynes KF Chemical and behavioral evidence for a third pheromone component in a North American population of the black cutworm moth, *Agrotis ipsilon* J Chem Ecol 1998 24 999 1011 10.1023/A:1022398318465

[B46] Picimbon JF Gadenne C Bécard JM Clément JL Sreng L Sex pheromone of the French black cutworm moth, *Agrotis ipsilon* (Lepidoptera: Noctuidae): identification and regulation of a multicomponent blend J Chem Ecol 1997 23 211 230

[B47] Wakamura S Struble DL Matsuura H Sato M Kegasawa K Sex pheromone of the black cutworm moth, *Agrotis ipsilon* Hufnagel (Lepidoptera: Noctuidae): attractant synergist and improved formulation Appl Entomol Zool 1986 21 299 304

[B48] Lienard MA Strandh M Hedenström E Johansson T Löfstedt C Key biosynthetic gene subfamily recruited for pheromone production prior to the extensive radiation of Lepidoptera BMC Evol Biol 2008 8 270 10.1186/1471-2148-8-270 18831750 PMC2584044

[B49] Dallerac R Labeur C Jallon JM Knipple DC Roelofs WL Wicker-Thomas C A Δ^9^ desaturase gene with a different substrate specificity is responsible for the cuticular diene hydrocarbon polymorphism in *Drosophila melanogaster* Proc Natl Acad Sci USA 2000 97 9449 9454 10.1073/pnas.150243997 10920187 PMC16884

[B50] Rafaeli A Mechanisms involved in the control of pheromone production in female moths: recent developments Entomol Exp Appl 2005 115 7 15 10.1111/j.1570-7458.2005.00292.x

[B51] Lienard MA Löfstedt C Functional flexibility as a prelude to signal diversity?: role of a fatty acyl reductase in moth pheromone evolution Commun Integr Biol 2010 3 6 586 588 10.4161/cib.3.6.13177 21331247 PMC3038071

[B52] Fang NB Teal PEA Tumlinson JH PBAN regulation of pheromone biosynthesis in female tobacco hornworm moths, *Manduca sexta* (L.) Arch Insect Biochem Physiol 1995 29 35 44 10.1002/arch.940290105

[B53] Luxová A Svatoš A Substrate specificity of membrane-bound alcohol oxidase from the tobacco hornworm moth (*Manduca sexta*) female pheromone glands J Mol Catal B-Enzym 2006 38 37 42 10.1016/j.molcatb.2005.10.006

[B54] Ishida Y Leal WS Rapid inactivation of a moth pheromone Proc Natl Acad Sci USA 2005 102 14075 14079 10.1073/pnas.0505340102 16172410 PMC1216831

[B55] Merlin C Rosell G Carot-Sans G François MC Bozzolan F Pelletier J Jacquin-Joly E Guerrero A Maïbèche-Coisne M Antennal esterase cDNAs from two pest moths, *Spodoptera littoralis* and *Sesamia nonagrioides*, potentially involved in odourant degradation Insect Mol Biol 2007 16 73 81 10.1111/j.1365-2583.2006.00702.x 17257210

[B56] Merlin C François MC Bozzolan F Pelletier J Jacquin-Joly E Maïbèche-Coisne M A new aldehyde oxidase selectively expressed in chemosensory organs of insects Biochem Biophys Res Commun 2005 332 4 10 10.1016/j.bbrc.2005.04.084 15896291

[B57] Pelletier J Bozzolan F Solvar M François MC Jacquin-Joly E Maïbèche-Coisne M Identification of candidate aldehyde oxidases from the silkworm *Bombyx mori* potentially involved in antennal pheromone degradation Gene 2007 404 31 40 10.1016/j.gene.2007.08.022 17904312

[B58] Rybczynski R Reagan J Lerner MR A pheromone-degrading aldehyde oxidase in the antennae of the moth *Manduca sexta* J Neurosci 1989 9 1341 1353 2703880 10.1523/JNEUROSCI.09-04-01341.1989 PMC6569867

[B59] Maïbèche-Coisne M Nikonov AA Ishida Y Jacquin-Joly E Leal WS Pheromone anosmia in a scarab beetle induced by in vivo inhibition of a pheromone-degrading enzyme Proc Natl Acad Sci USA 2004 101 11459 11464 10.1073/pnas.0403537101 15277687 PMC509178

[B60] Maïbèche-Coisne M Merlin C François MC Porcheron P Jacquin-Joly E P450 and P450 reductase cDNAs from the moth *Mamestra brassicae*: cloning and expression patterns in male antennae Gene 2005 346 195 203 15716002 10.1016/j.gene.2004.11.010

[B61] Wojtasek H Leal WS Degradation of an alkaloid pheromone from the pale-brown chafer, *Phyllopertha diversa* (Coleoptera: Scarabaeidae), by an insect olfactory cytochrome P450 FEBS Lett 1999 458 333 336 10.1016/S0014-5793(99)01178-3 10570935

[B62] Durand N Carot-Sans G Chertemps T Montagné N Jacquin-Joly E Debernard S Maïbèche-Coisne M A diversity of putative carboxylesterases are expressed in the antennae of the noctuid moth *Spodoptera littoralis* Insect Mol Biol 2010 19 87 97 20002215 10.1111/j.1365-2583.2009.00939.x

[B63] Rogers ME Jani MK Vogt RG An olfactory-specific glutathione-S-transferase in the sphinx moth *Manduca sexta* J Exp Biol 1999 202 1625 1637 10333508 10.1242/jeb.202.12.1625

[B64] Zhou JJ He XL Pickett JA Field LM Identification of odorant-binding proteins of the yellow fever mosquito *Aedes aegypti*: genome annotation and comparative analyses Insect Mol Biol 2008 17 147 163 10.1111/j.1365-2583.2007.00789.x 18353104

[B65] Zhou JJ Kan YC Antoniw J Pickett JA Field LM Genome and EST analyses and expression of a gene family with putative functions in insect chemoreception Chem Senses 2006 31 453 465 10.1093/chemse/bjj050 16581978

[B66] Clement SL Show ED Way MO Black cutworm pheromone trapping in strawberries Calif Agric 1982 36 20 21

[B67] Rings RW Arnold FJ Keaster AJ Musick GJ A worldwide annotated bibliography of the black cutworm, *Agrotis ipsilon* (Hufnagel) Ohio Agric Res Dev Cent Res Circ 1974 198 1 106

[B68] Han ZJ Toxicological responses and resistances of the black cutworm, *Agrotis ypsilon* (Rottemberg), to several groups of insecticides Acta Phytophylacica Sinica 1986 13 125 130

[B69] Gemeno C Haynes KF Periodical and age-related variation in chemical communication system of black cutworm moth, *Agrotis ipsilon* J Chem Ecol 2000 26 329 342 10.1023/A:1005468203045

[B70] Swier SR Rings RW Musick GJ Age-related calling behavior of the black cutworm, *Agrotis ipsilon* Ann Entomol Soc Am 1977 70 919 924

[B71] Xiang YY Yang MF Li ZZ Calling behavior and rhythms of sex pheromone production in the black cutworm moth in China J Insect Behav 2010 23 35 44 10.1007/s10905-009-9193-0

[B72] Lassmann T Hayashizaki Y Daub CO TagDust-a program to eliminate artifacts from next generation sequencing data Bioinformatics 2009 25 2839 2840 10.1093/bioinformatics/btp527 19737799 PMC2781754

[B73] Chou HH Holmes MH DNA sequence quality trimming and vector removal Bioinformatics 2001 17 1093 1104 10.1093/bioinformatics/17.12.1093 11751217

[B74] Chen YA Lin CC Wang CD Wu HB Hwang PI An optimized procedure greatly improves EST vector contamination removal BMC Genomics 2007 8 416 10.1186/1471-2164-8-416 17997864 PMC2194723

[B75] Meyer E Aglyamova GV Wang S Buchanan-Carter J Abrego D Colbourne JK Willis BL Matz MV Sequencing and *de novo* analysis of a coral larval transcriptome using 454 GSFlx BMC Genomics 2009 10 219 10.1186/1471-2164-10-219 19435504 PMC2689275

[B76] Chevreux B Pfisterer T Drescher B Driesel AJ Müller WEG Wetter T Suhai S Using the miraEST assembler for reliable and automated mRNA transcript assembly and SNP detection in sequenced ESTs Genome Res 2004 14 1147 1159 10.1101/gr.1917404 15140833 PMC419793

[B77] Huang XQ Madan A CAP3: a DNA sequence assembly program Genome Res 1999 9 868 877 10.1101/gr.9.9.868 10508846 PMC310812

[B78] Altschul SF Madden TL Schäffer AA Zhang JH Zhang Z Miller W Lipman DJ Gapped BLAST and PSI-BLAST: a new generation of protein database search programs Nucleic Acids Res 1997 25 3389 3402 10.1093/nar/25.17.3389 9254694 PMC146917

[B79] Anderson I Brass A Searching DNA databases for similarities to DNA sequences: when is a match significant? Bioinformatics 1998 14 349 356 10.1093/bioinformatics/14.4.349 9632830

[B80] Conesa A Götz S García-Gómez JM Terol J Talón M Robles M Blast2GO: a universal tool for annotation, visualization and analysis in functional genomics research Bioinformatics 2005 21 3674 3676 10.1093/bioinformatics/bti610 16081474

[B81] Ye J Fang L Zheng HK Zhang Y Chen J Zhang ZJ Wang J Li ST Li RQ Bolund L Wang J WEGO: a web tool for plotting GO annotations Nucleic Acids Res 2006 34 W293 W297 10.1093/nar/gkl031 16845012 PMC1538768

[B82] Strandh M Johansson T Ahrén D Löfstedt C Transcriptional analysis of the pheromone gland of the turnip moth, *Agrotis segetum* (Noctuidae), reveals candidate genes involved in pheromone production Insect Mol Biol 2008 17 73 85 10.1111/j.1365-2583.2008.00782.x 18237286

[B83] Bendtsen JD Nielsen H von Heijne G Brunak S Improved prediction of signal peptides: signalP 3.0 J Mol Biol 2004 340 783 795 10.1016/j.jmb.2004.05.028 15223320

[B84] Thompson JD Gibson TJ Plewniak F Jeanmougin F Higgins DG The CLUSTAL_X windows interface: flexible strategies for multiple sequence alignment aided by quality analysis tools Nucleic Acids Res 1997 25 4876 4882 10.1093/nar/25.24.4876 9396791 PMC147148

[B85] Livak KJ Schmittgen TD **Analysis of relative gene expression data using real-time quantitative PCR and the 2-**^**ΔΔCt **^**method.** Methods 2001 25 402 408 10.1006/meth.2001.1262 11846609

