# Supplementary figures and images for: Identification of genes expressed in the sex pheromone gland of the black cutworm Agrotis ipsilon with putative roles in sex pheromone biosynthesis and transport
Source: BMC Genomics. 2013 Sep 22;14:636. doi: 10.1186/1471-2164-14-636 (PMC3849270; doi:10.1186/1471-2164-14-636)

**Additional file 1.**


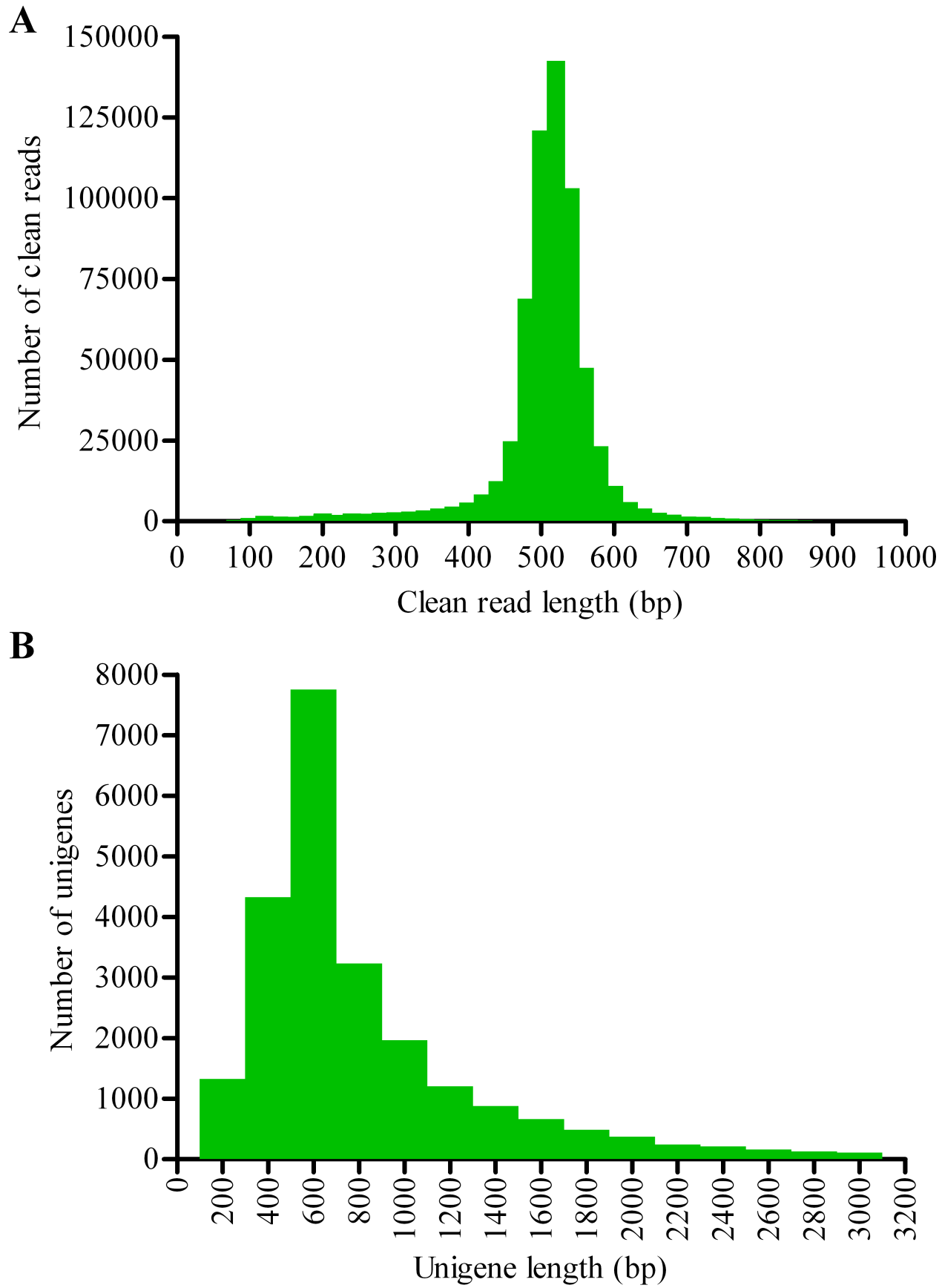

Supplement: Additional file 1 — Size distribution of the clean reads (A) and the assembled unigenes (B). [file 1471-2164-14-636-S1.docx]
