# Supplementary material for: Identification of genes expressed in the sex pheromone gland of the black cutworm Agrotis ipsilon with putative roles in sex pheromone biosynthesis and transport
Source: BMC Genomics. 2013 Sep 22;14:636. doi: 10.1186/1471-2164-14-636 (PMC3849270; doi:10.1186/1471-2164-14-636)
Supplement: Additional file 4 — Primers used for RT-PCR analysis of enzyme genes of the A. ipsilon PG. [file 1471-2164-14-636-S4.docx]

| Primer name | Forward primer (5’-3’) | Reverse primer (5’-3’) |
| --- | --- | --- |
| Acetyl CoA Carboxylase |  |  |
| Unigene_2338 | CAGCTGTCCACGATTTGCTA | TGAGCTCGTTGAGTGTGGTC |
| Unigene_6244 | TCAAAATGGCTGAGCAACAG | GTCTGTTTGGCCGTGGTATT |
| Fatty acid synthase |  |  |
| Unigene_18120 | AGAAGATGGACCCCAGGATT | ATCTCCTCCGTCTCCGAGTT |
| Desaturases |  |  |
| Unigene_65 | CACGGTGATTCCGGTCTACT | AGGCTTGATGCTCTTGTCGT |
| Unigene_741 | TTTTGGAAAATGGCTCAAGG | GCTAACACGACGAGCATGAA |
| Unigene_780 | GGTCGTGGGTCCTCTATGAA | GAAACACGTCGTCCACATTG |
| Unigene_10494 | GCTTTTCAGAATGGCTCCAG | CATTGCGCGTTAAATTCCAT |
| Unigene_15401 | GTGCGAGATCACAGAGTCCA | AAGCAGAGGATTGGCATCAC |
| Fatty acyl reductase |  |  |
| Unigene_163 | GAGAGCACCATGATGCAGAA | ACTTCAGCGCTTCGTTCATT |
| Unigene_1098 | ACGAGGTGGACGTGATCTTC | TTTGTGGGCAGTTGCTGTAG |
| Unigene_2537 | GCTGAACACGAAGTGGTGAA | TCACGACAACATCCACTGGT |
| Unigene_3905 | AACAGACGTTTCCGATGGAC | ACCGGTTCGACGTAAGACAC |
| Unigene_4078 | CTGAACGTGAGAGGCACAAA | TCTCCTCATCCATCCACTCC |
| Unigene_4302 | GCAAACTGCACCTCTTCACA | TGGTCTGGGGATCCAAATTA |
| Unigene_6708 | TGTGGTACCGGTAGACGTGA | AACTCCTGGACGTGGATACG |
| Unigene_7344 | CGGATCAAGGTCTTCATACACATATCC | TGTACCTGATGCTCAGCCGCGA |
| Unigene_8541 | GAATATAGAGGAAGTTCGCCACCTCTG | GCAGATGGCGAGACCATCGACTAG |
| Unigene_11561 | CATGTAGCTGCTAGTGTTAGGTTCG | GATACAGCACCTCCTCGATAGGATC |
| Unigene_12329 | TATCGTGAGCCATCAGCCATG | CTTCTTATCCCTCATGAGAAGGTGG |
| Unigene_12652 | TCCAAATATTCCAGAGTTTTACAAAGG | GCCAGCGATGACAAATACTTTTG |
| Unigene_15351 | CGTGGACCCAGATCACGTAATG | CATTACACAGCATGGTTCTGATGAC |
| alcohol oxidase/ dehydrogenase |  |  |
| Unigene_195 | AATGGCACAGTGGGACGCAAA | AGTCGATCATTACGCCTGCATTGT |
| Unigene_307 | CACGCGTGAAGATTTCGACATAG | CGTTTGTATCAATGACGCAGTAATC |
| Unigene_397 | GTAGTCGTAATAACTGGTGGAGCCG | GATGTCGATGGTCTTCTTGAGGAGA |
| Unigene_7733 | TTCGTGAAGATGTTCGAAATTAGAGAT | CATTGTCGTATACCGCATCCAGTT |
| Unigene_10714 | AAGATCCTAGGATGTCGAGTCATTGG | CTGCCGTATACGTTCATCTGGGAC |
| aldehyde reductase |  |  |
| Unigene_1256 | ACCTGTATAGAGTGGAACCGGAAGTC | CGCCAGGTTTCCAAGTAGTCGAT |
| Unigene_1274 | ATGGACGCACTTGTCCAGTGTTG | TCGGTGGAAGGTGCACCAGAG |
| Unigene_1735 | CTAACAGGACATGCAGCAAATCCA | ATCGTCGATGCCCTTTCCAA |
| Unigene_1774 | ATGGCGCGAGTTCCTACAATCA | CGATGGAAGGTGTTCCATAGCTT |
| Unigene_3134 | ATGGACGAGAGATGCCCATGA | TCCTCTTCAGCATGCCGGTC |
| Unigene_4806 | ATACGTATCACAGACCGGATTTAGTGG | CCGATGCTCTTAACGAGGCCTT |
| Unigene_5103 | ATAGCTGAAGGCGTTGTGAGTAGAGA | TGATCAGTCTGTCCAGCTGCGTG |
| Unigene_7337 | ATGGGAACGCTATACCAGCGTTG | GGAAGCTTGGTGACTACGAAGACATT |
| Unigene_7554 | GCAAGATATGAGTCCACCACTGTAAG | GGCTCCAATCACGTGCTGGAT |
| Unigene_9245 | AGATCCGCTGAAGGAGTTGTAACC | CAGGCCTAGTCTGTGCATGTCTTC |
| Unigene_9786 | ATCGCTCACCGTAGCGAAGGTA | CCCTCTTGAGTCCCTCCTTGAATT |

**Additional file 4**. Primers used for RT-PCR analysis of enzyme genes of the *A. ipsilon* PG.

**Supplementary Table 1**. **Continued.**

| Primer name | Forward primer (5’-3’) | Reverse primer (5’-3’) |
| --- | --- | --- |
| Acetyltransferase |  |  |
| Unigene_173 | AAGGACGTGTACCCTTCAGACCTG | CTGAGCACTGTTGACGACAGCTT |
| Unigene_407 | GTCTCGGAGTACGGTGGTTTCC | TAGCCACGTGGAACGCTCCC |
| Unigene_553 | ATGTCCGCAGCAGCTAAAGGTAT | CAGCATAACGTGACGAGGTAGGTAGA |
| Unigene_2015 | CAAGATGAACATCCGGTGTGCTC | CTAGGCGACGATGAGACCTCTTC |
| Unigene_15362 | GAGCTGTTCATAACTGCGATGGC | CGAATATTGCGGCTTGTCTAGCTG |
| Reference gene |  |  |
| β-actin | ACCACACCTTCTACAACGAGCTG | AGCGCGTATCCCTCGTAGATG |
