# Supplementary material for: Identification of genes expressed in the sex pheromone gland of the black cutworm Agrotis ipsilon with putative roles in sex pheromone biosynthesis and transport
Source: BMC Genomics. 2013 Sep 22;14:636. doi: 10.1186/1471-2164-14-636 (PMC3849270; doi:10.1186/1471-2164-14-636)
Supplement: Additional file 5 — Primers used in real-time PCR for determination expression level of target genes. [file 1471-2164-14-636-S5.docx]

| Primer name | Forward primer (5’-3’) | Reverse primer (5’-3’) |
| --- | --- | --- |
| Acetyl CoA Carboxylase |  |  |
| Unigene_2338 | GTATCTCGGCAAGGCTAA | CTGATGATGGAACGGATGA |
| Unigene_6244 | TTGTTATTAAGTTATTGGAGTGG | TTCCTTAGCCTCTTCTGA |
| Fatty acid synthase |  |  |
| Unigene_18120 | TCGTGTGGAGAACTATGG | TCGTCGGAACTGAATGTT |
| Desaturases |  |  |
| Unigene_65 | TTACTCTTCGCATATCTTC | CCTGACATCACATACAAG |
| Unigene_741 | GTGTTAGCAGAGTTAGGA | AGTTCAATACCATCAGGAT |
| Unigene_780 | TGGAAGATAGTATGGAGAA | ATATACAAGAACACAGCAA |
| Unigene_10494 | ATGGAAATGTGTGGTGAAG | CTGTATTGTTATTCTCGTAATGTG |
| Unigene_15401 | ATGTCCGACCTGGAGAAAG | GGATTATGAAGCAGAGGATTGG |
| Fatty acyl reductase |  |  |
| Unigene_163 | GCACGCTCAGAGAAGAAT | CATAACAGAACGGTCAGGAA |
| Unigene_1098 | GACGAGAAGTTCTATGAC | TGTAATGTTGTTGATGGT |
| Unigene_2537 | TACGATGTATGCCTGATG | TTCTGGAATATGTCTGTTGA |
| Unigene_3905 | GTATCATCATCATTGCCTAC | TAGTATGTCGGTCATCAC |
| Unigene_4078 | GTCCATCGTGATTCCAAT | ACCAATAAGTAATCCAGTAGG |
| Unigene_4302 | GAATGAGCCTGATAACTG | GTAACTTCTTCACATTGTCT |
| Unigene_6708 | GCTCTGTTCTTCACTCAC | GTTGATGCGTTTCTGGAT |
| Unigene_7344 | GTAATCAACTTCTTCACAT | TAGGTATTCTGTCCAATC |
| Unigene_8541 | CTGTGTGGTTCATTTCTGT | ACTTGTGGATCTTACGGTATA |
| Unigene_11561 | AAGTCATGGAATTGGCTAA | CTGTTGGTGTTGGAGTAT |
| Unigene_12329 | CCTAATACTTACACTTTCAC | ATCACGATAGTTGGTCTA |
| Unigene_12652 | AGACAGATTGAACGAACT | CAGCGATGACAAATACTTT |
| Unigene_15351 | TATAGGAATGCTTGAAGGAAG | TTCTGATGACCGTTGTTC |
| alcohol oxidase/ dehydrogenase |  |  |
| Unigene_195 | AAGAGAGAATCAGCAGTT | TTATCAATAGCCGCAGTA |
| Unigene_307 | GGTGTGGATTGTTACTTC | ATGTTATTCTCGTTGTATGA |
| Unigene_397 | ATCTGCCAACTATTTCCAA | TCTTCTTGAGGAGACGAT |
| Unigene_7733 | GTCACAACTAACGAACTG | ACTTCTTATCAAGGCTGTA |
| Unigene_10714 | AAAGCGGAAAGCGACAAT | TACGGTTGGCATATCTATTCAC |
| aldehyde reductase |  |  |
| Unigene_1256 | GCAAGATTATCAGAACTAC | TTAAGTCCATTCTTCCAA |
| Unigene_1274 | CAGAATGAGAAGGAAGTT | CGAAGTTATGAAGATGTC |
| Unigene_1735 | AGGTCCATCCATCAAGAAC | TGTATGCTGTGACTGCTATT |
| Unigene_1774 | AACCTTAACCTGAAGTATT | ACGCTATGTTATCTGAAT |
| Unigene_3134 | GGTGGTGTTATCATTGAA | GCTTCCTCATTCTTATTCT |
| Unigene_4806 | TTGTCTGATCTCGGATTG | CCATTAGCATCTGAAGGA |
| Unigene_5103 | TGTGTATTGTAACTAAGC | CAGATAGATGTCCAGATA |
| Unigene_7337 | ATGTGTTGTCTTCCTTGA | ACAGTAGAATGTATGAGGTA |
| Unigene_7554 | TAACAGATGAGGAATATAACAGA | AATGCCAACACAGGTATT |
| Unigene_9245 | CTATACAGATTGAGGTCCAT | CCATCACTATCATATCATTAGC |
| Unigene_9786 | CGATGTGTTTGACTTTAC | AAGGATAGTATGGATGGT |

**Additional file 5.** Primers used in real-time PCR for determination expression level of target genes.

**Supplementary Table 2**. **Continued.**

| Primer name | Forward primer (5’-3’) | Reverse primer (5’-3’) |
| --- | --- | --- |
| Acetyltransferase |  |  |
| Unigene_173 | TCGGAGTCAATTTAGGAGTA | TGTGGCATAGTGTTGTTG |
| Unigene_407 | CAGTTCCATTGCCAGATA | CACCGAAGACCAAGTATC |
| Unigene_553 | GACTCACTGACTCATACT | AAGTTATCAACCTCATCC |
| Unigene_2015 | GCCTCAACTCAGTTATGT | GTCCTCACCATCTTCTTC |
| Unigene_15362 | GAGAATGGAACTATAACAG | ATCAGCATAACCAATAAC |
| Odorant binding proteins |  |  |
| Unigene_520 | ACATCTTCACCTGTCTCA | AGTTTGTCGCACTCATTT |
| Unigene_2120 | GAGTTAGCAACGATGACATT | ATCCGAAGAAGCAAGGTT |
| Unigene_6517 | ACTAATGCCAAGAAGGTA | GCTTACAGTTCCATAATACA |
| Unigene_8860 | GGACAAGACCAAGGAATT | ACTTCTTCATCGTTCACTT |
| Unigene_15218 | GAAGGTTCTGGAAGGATG | TAGCATTAGCGTGGTAAC |
| Unigene_15711 | AGGCAACATCTGGACATC | AATCTGGACATTTCTTCAACATAG |
| Unigene_15861 | AAACGGTCTCATCAAACTTAGGAA | GCACTCTGTAGCAACTTCTTCA |
| Chemosensory proteins |  |  |
| Unigene_468 | CCAGAGGAAGAGAAGTAT | AAGTAAGCAGTCACAATAG |
| Unigene_1704 | CTTATCCGTACCGTAGTC | ATCTTCCTTCTTGACCAG |
| Unigene_1767 | GCTGACTACTGGAATGAG | TTGAGTTCCTTCTCGTATT |
| Unigene_2047 | ACTCGTTGTGCTAAATGTA | CAGTTCCTTCCAGATGTC |
| Unigene_6052 | CACAAGAAACTAAGCAGATACA | GCGTACTGGCGAACTATT |
| Unigene_15000 | CTTGTGGAAGGAGTTGTC | GATTCAATTTGGGTCTTGTATT |
| Unigene_15308 | GAAGTTCGTCTTGCTATT | ATCTCATCCAGGTCAATA |
| Unigene_18519 | TACGACCACATCAATATC | CTTCAGGAGTACATCTTC |
| Candidate esterase genes |  |  |
| AipsCXE1 | CATCTTACGAGGAGGATT | TATGGCTTGTCTGTAGTT |
| AipsCXE2 | GGAGTTACGACCATCATTG | AGTAGCGTATCCTATTAGAAGT |
| AipsCXE3 | TGCTATAATACCAGGAAG | TGAATGAAGACCATAACT |
| AipsCXE4 | ATTACTGGATAGAACTGAT | AACTGGTAACTTCTTGTA |
| AipsCXE5 | TGGCAACAACTCTCACTA | ACAGGTAGATCAGATCATCAT |
| AipsCXE6 | ATATTAGTCTGGTTCCAT | GTCTATAATTCACTGTTATG |
| AipsCXE7 | GTTCAAGAGGATTGCCAGAG | ATGGACAGACACGAGGAC |
| AipsCXE8 | TATCCTTACTTGTTGCTA | TTACTTGCTACTGATACT |
| AipsCXE9 | AAGTGCCGTGTATTATTGGTAG | GGAACAAGAAGCCTGACATT |
| AipsCXE10 | ACCAATGAAGACTGCCTATT | AACCGCCATGAATGTAGAA |
| AipsCXE11 | CGTAGTGATTGTCAGTAT | TTAGTGCTTGAACTTGAT |
| AipsCXE12 | TCAATAGGTATATGGAGCAAGA | CGGTCACATTCTGGTAGT |
| AipsCXE13 | AATGTCGTTGAGGTTATTCTT | TCGTATAGTCGGGTTTGG |
| AipsCXE14 | TAAGTCTATTGACAATGGAT | AAGGAGATTCTGTAAGGA |
| AipsCXE15 | TAACTTATTCTGTGTCTGA | CACTTGATTATGTTGTCTAT |
| AipsCXE16 | CGGGATTCGGAAAGTTGT | GCATCGCCATCAAGTTCA |
| AipsCXE20 | TGTTCAATAGAGCCATCAT | TGTTGTGCTAGGAGACTA |
| Reference genes |  |  |
| β-actin | GATGTTGACATTCGTAAG | ATCTTGATCTTCATTGTG |
| RPS3 | AAGTCCATGAAGTTCGTA | GTACTCCTTGTCTTAGCA |
